# CircRSU1 Activates the hnRNPA1/HIF‐1α/CD24 Signaling Axis, Promoting Stemness Features of Hepatocellular Carcinoma

**DOI:** 10.1002/advs.202522424

**Published:** 2026-02-12

**Authors:** Shuting Xue, Danduo Wei, Yongzhi Zhao, Jinchang Pan, Hanyuan Zhang, Niya Liu, Jianjuan Zhang, Jiaxin Liu, Jianping Jin, Mu Xiao, Xinhua Feng, Aifu Lin, Lei Zhao, Stephanie Roessler, Junfang Ji

**Affiliations:** ^1^ The MOE Key Laboratory of Biosystems Homeostasis & Protection Zhejiang Key Laboratory of Molecular Cancer Biology Life Sciences Institute Zhejiang University Hangzhou Zhejiang Province China; ^2^ Center For Life Sciences Shaoxing Institute Zhejiang University Shaoxing Zhejiang Province China; ^3^ Cancer Center Zhejiang University Hangzhou Zhejiang Province China; ^4^ College of Life Sciences Zhejiang University Hangzhou Zhejiang Province China; ^5^ Shandong Cancer Hospital and Institute Shandong Cancer Hospital of Shandong First Medical University Jinan Shandong Province China; ^6^ Institute of Pathology University Hospital Heidelberg Heidelberg Germany

**Keywords:** CD24, circRSU1, hepatocellular carcinoma, HIF‐1α, hnRNPA1

## Abstract

Hepatocellular carcinoma (HCC) is a highly lethal malignancy with obvious heterogeneity features. This study aims to identify circRNAs with key roles in promoting HCC malignancy and stemness properties. Through circRNA profiling comparison in HCC patients and functional screening in HCC cells, circRSU1 emerges as the top candidate. It exhibits a significant higher expression level in tumor tissues compared to adjacent non‐tumor liver tissues from HCC patients. Functionally, circRSU1 promotes a spectrum of HCC malignant phenotypes both in vitro and in vivo, including an enrichment of CD24^positive^ cancer stem cell population. Mechanistically, circRSU1 interacts with heterogeneous nuclear ribonucleoprotein A1 (hnRNPA1) via two RNA motifs on circRSU1 and two RNA‐binding domains on hnRNPA1. This interaction increases hnRNPA1 protein level via reducing its proteasomal degradation. Furthermore, hnRNPA1 enhances HIF‐1α protein translation via binding to its internal ribosome entry site (IRES), which subsequently increases the CD24^positive^ cell population. Additionally, circRSU1 further enhances this process not only through increasing the hnRNPA1 protein level, but also through enhancing the interaction of hnRNPA1 with *HIF1A* IRES, consequently augmenting the CD24^positive^ cell population and the associated malignancy/stemness features of HCC cells. Together, circRSU1 activates the hnRNPA1/HIF‐1α/CD24 signaling axis, leading to the increased HCC malignancy and stemness features.

## Graphical abstract

This study reveals circRSU1 with important oncogenic roles in hepatocellular carcinoma (HCC). CircRSU1, highly abundant in HCC, interacts with and stabilizes hnRNPA1 from ubiquitination and degradation. This stabilization facilitates hnRNPA1 binding to the internal ribosome entry site of HIF1A to increase HIF‐1α protein translation. Subsequently, the increased HIF‐1α enhanced the CD24‐positive cancer stem cell population and HCC malignancy features.

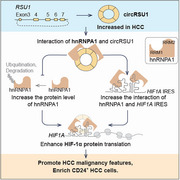

**Abbreviations**
ASO: Antisense Oligonucleotide
BSJ: Back‐Splicing Junction
CSC: Cancer Stem Cell
circRNA: Circular RNA
CHX: Cycloheximide
FFPE: Formalin‐Fixed and Paraffin‐Embedded
GSEA: Gene Set Enrichment Analysis
GPI: Glycosyl‐phosphatidylinositol
HCC: Hepatocellular carcinoma
hnRNPA1: Heterogeneous Nuclear Ribonucleoprotein A1
HDTV: Hydrodynamic tail vein injection
HIF‐1α: Hypoxia‐Inducible Factor 1 Alpha
IRES: Internal Ribosome Entry Site
ORF: Open Reading Frame
PCR: Polymerase Chain Reaction
RBP: RNA Binding Protein
RIP: RNA Immunoprecipitation
RRM: RNA Recognition Motif
RGG: Arg‐Gly‐Gly containing
siRNA: Small Interfering RNA

## 1 Introduction

Primary liver cancer is the seventh most common cancer and the third leading cause of cancer‐related mortality worldwide [1, 2]. Hepatocellular carcinoma (HCC) accounts for ∼80% of these cases, with a five‐year survival rate of less than 20% [3]. Surgical interventions, including tumor resection and liver transplantation, are the principal curative treatments for HCC. However, the majority of patients are diagnosed with HCC at an advanced stage, missing the opportunity for surgical intervention so that presenting the poor prognosis [4]. In these patients, HCC tumors often exhibit the activated malignancy and stemness molecular signatures [5, 6]. The existence or enrichment of cancer stem cells (CSCs) is responsible for HCC malignancy and stemness features. Several CSC biomarkers have been identified and evaluated in HCC, such as CD24, EpCAM, CD133, and CD90 [7, 8, 9, 10, 11]. These marker‐positive HCC cells present the malignancy and stemness features, and HCC patient carrying these cells show poor prognosis. To improve HCC outcome, it is thus crucial to thoroughly investigate the regulatory mechanisms of HCC malignancy and stemness features with a focus on these CSC marker‐positive cells.

Circular RNAs (circRNAs) are a class of endogenous RNAs characterized by a covalently closed circular structure, and they are involved in a wide spectrum of signaling molecular pathways [12]. It functions in many human diseases, including cancer, via sponging miRNAs, interacting with proteins, modulating gene expression, and potentially encoding proteins [13]. In HCC, several circRNAs have been identified with notable roles, such as cSMARCA5 in suppressing HCC [14] and circRNA‐SORE in sorafenib resistance [15]. However, very limited information was known about whether and how circRNAs functioned in regulating the CSC marker‐positive HCC cell populations. Recently, it has been reported that circCDYL is highly expressed in early HCC and knocking down circCDYL in vitro significantly reduces the EpCAM^positive^ cell populations [16]. EpCAM is an identified hepatic CSC marker, and the enriched EpCAM^positive^ cells present the malignancy and stemness features [10, 11]. In this case, it is likely that circRNAs are important in regulating HCC malignancy and stemness features, especially regulating a particular CSC population.

In this study, we aimed to identify circRNAs with high abundance in HCC tumors and their roles in regulating HCC stemness and malignancy features. Through systematic screening, circRSU1 emerged as an important candidate. Subsequent cellular and molecular assays revealed that it significantly enhanced HCC malignancy and stemness features including the specific enrichment of CD24^positive^ cell population. This effect was mediated via the circRSU1/hnRNPA1/ HIF‐1α signaling axis. In sum, circRSU1 enhances HCC malignancy and stemness features, and may serve as a potential therapeutic target for HCC patients enriched with CD24^positive^ CSC population.

## 2 Results

### 2.1 circRSU1 was a Highly Abundant Circular RNA in HCC and Promoted HCC Spheroid Formation

**FIGURE 1:**
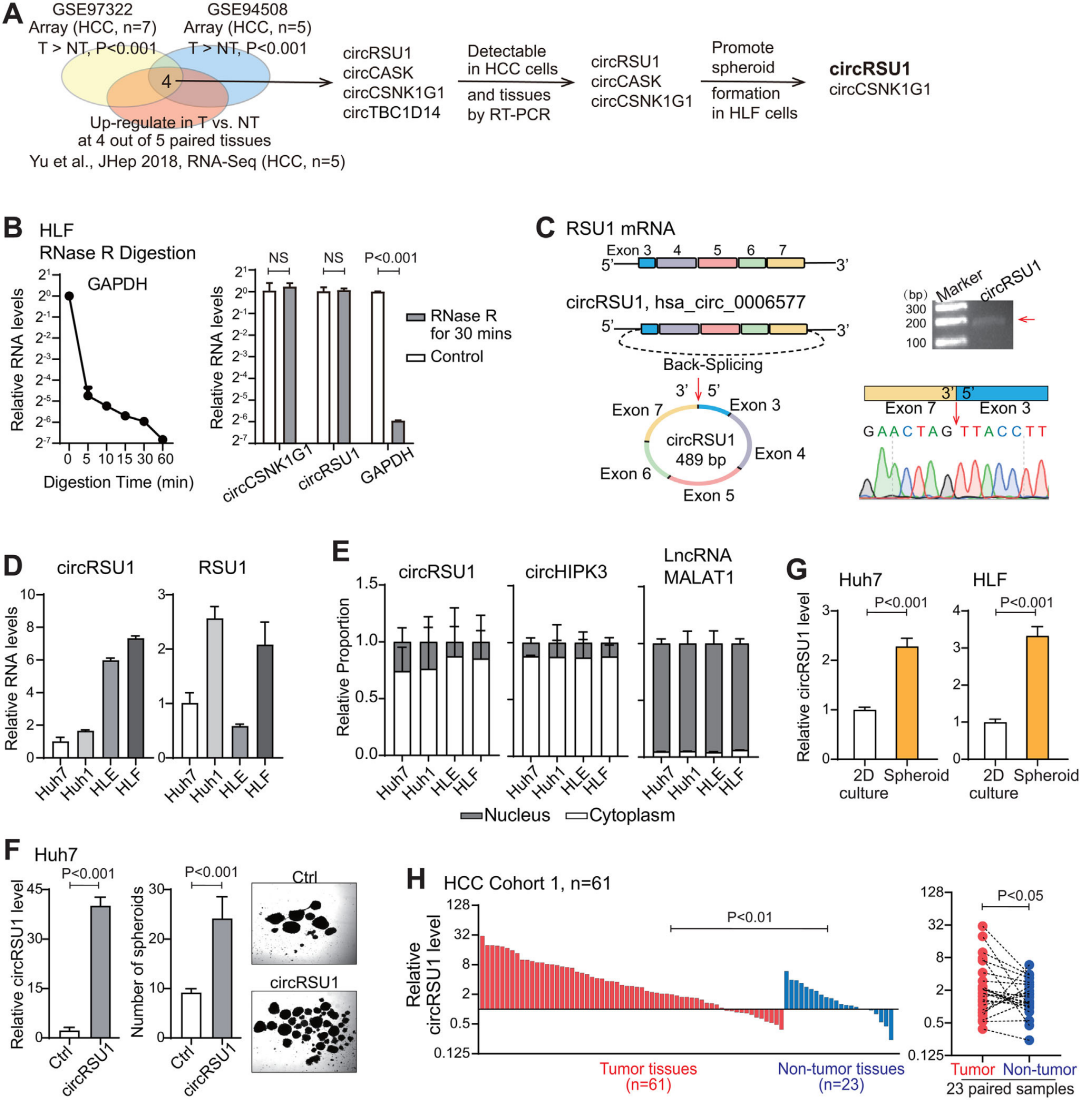
CircRSU1 is highly expressed in HCC tumors and promotes spheroid formation in HCC cells. (A) The flowchart of our screening strategy to identify circRNAs with high abundance in HCC tumors and roles in promoting stemness features. (B) GAPDH mRNA level in HLF cells at different time points following RNase R treatment (left panel); the mRNA levels of circCSNK1G1, circRSU1, and GAPDH in HLF cells with 30 min of RNase R treatment (right panel). (C) Schematic representation of back‐splicing of RSU1 gene exon 3 to exon 7, and BSJ site of circRSU1 (left panel). Agarose gel electrophoresis of circRSU1 PCR products and Sanger sequencing results of BSJ site (right panel). (D) Expression level of circRSU1 and its host mRNA RSU1 in HCC cell lines. (E) Subcellular localization of circRSU1 in four HCC cell lines via PCR examination of its expression level. CircHIPK3 and LncRNA MALAT1 served as positive controls for cytoplasmic and nuclear fractions, respectively. (F) Spheroid formation with low‐attachment plates in Huh7 cells transfected with control or pCIR‐circRSU1 vector. (G) CircRSU1 expression level in HLF and Huh7 cells under standard 2D culture conditions and spheroid culture conditions. (H) CircRSU1 expression in tumor and adjacent non‐tumor FFPE tissues from HCC Cohort 1 (n = 61, including 23 paired samples). (B, F, G, H) Student's t‐test was performed for statistical analysis.

To identify circRNAs with high abundance in HCC tumors and roles in regulating HCC stemness and malignancy features, we established an expression‐based and functional screening approach (Figure 1A). First, we compiled circRNA profiling data from three independent HCC circRNA datasets, i.e., GSE97322 [17], GSE94508 [18], and a circRNA dataset published in *Journal of Hepatology* [14]. Class comparison was performed within each dataset between tumors and non‐tumor tissues. Across all three datasets, four circRNAs presented higher levels in HCC tumor tissues compared to the adjacent non‐tumor liver tissues. Of these, three circRNAs were detectable in formalin‐fixed, paraffin‐embedded (FFPE) HCC tumor tissues (Figure S1A). Second, in HLF cells, two of the three candidate circRNAs significantly promoted spheroid formation, a measure of self‐renewal ability as one of key cancer stemness characteristics (Figure S1B,C). They are circRSU1 and circCSNK1G1. CircCSNK1G1 has been found to facilitate HCC cell proliferation and migration recently [19], suggesting the efficacy of our screening. CircRSU1 promoted the spheroid formation most significantly and its role in HCC has not yet been reported. Thus, we selected circRSU1 as the key candidate for further evaluation (Figure 1A).

The physical existence of circRSU1 in HCC cells was then confirmed. CircRNAs are resistant to degradation by exonuclease RNase R. Following the RNase R treatment, circRSU1 level remained unchanged, whereas the level of GAPDH as a negative control was noticeably reduced (Figure 1B). CircCSNK1G1 was used as a positive control. According to the circBank database, circRSU1 is predicted to arise from the back‐splicing of exons 3–7 of RSU1 gene, with a length of 489 nt. Consistently, the predicted back‐splicing junction (BSJ) site and sequence of circRSU1 were verified in HCC cells by Sanger sequencing (Figure 1C).

Among four HCC cell lines, circRSU1 showed a heterogeneous expression, with a relatively high level in HLF cells but a low level in Huh7 cells (Figure 1D; Figure S1D). Meanwhile, its expression was not parallel with its host mRNA RSU1. Subcellular fractionation of RNA from all four HCC cell lines indicated that circRSU1 was predominantly localized in the cytoplasm (Figure 1E). circHIPK3 and LncRNA MALAT1 were used as positive controls for cytoplasmic RNA and nuclear RNA, respectively [20, 21].

Moreover, consistent data was obtained that overexpressed circRSU1 in Huh7 cells significantly promoted spheroid formation (Figure 1F). Meanwhile, circRSU1 expression was significantly elevated in spheroids from 3D culture condition compared to cells of the 2D culture, in both Huh7 and HLF cells (Figure 1G). This result was comparable with the argument that cells growing as 3D spheroids retained a higher level of stemness compared to cells under the standard 2D culture condition [6, 11, 22]. Furthermore, in HCC Cohort 1 including 61 HCC cases, RT‐PCR results revealed that circRSU1 expression level was significantly higher in tumor tissues compared to adjacent non‐tumor tissues from HCC patients (Figure 1H). Together, circRSU1 was highly expressed in HCC tumor tissues and promoted HCC spheroid formation, a key cancer stemness feature.

### 2.2 Silencing circRSU1 Significantly Suppressed HCC Malignancy Features both In Vitro and In Vivo

**FIGURE 2:**
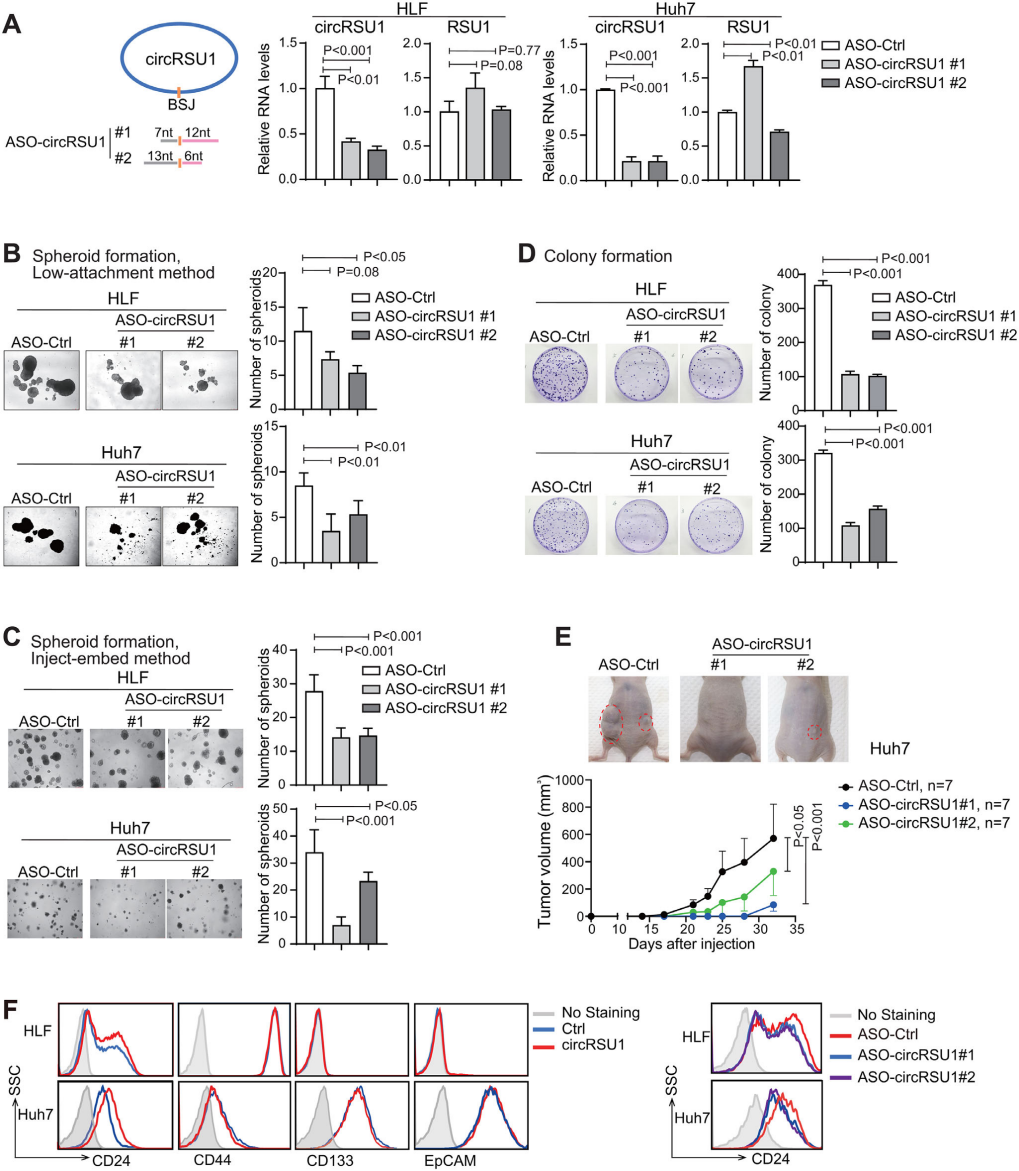
Silencing circRSU1 inhibits HCC malignancy and stemness features in vitro and in vivo. (A) Silencing efficiency of ASOs targeting circRSU1 was assessed in HLF and Huh7 cell lines. (B–D) Spheroid formation with low‐attachment plates (B), spheroid formation with inject‐embed method (C), and colony formation (D) were performed in HLF and Huh7 cells transfected with ASO‐control or ASOs targeting circRSU1. (A–D) Student's t‐test was performed. (E) Tumorigenicity assay was performed in Huh7 cells transfected with ASO‐control or ASOs targeting circRSU1. Male BALB/c nude mice were used and seven sites per group were injected. Tumor volume was assessed, and representative tumor images were shown. The two‐way ANOVA analysis was performed. (F) Flow cytometry was performed to assess the proportion of cells positive for CSC markers in Huh7 and HLF cells with the altered level of circRSU1.

To explore the role of circRSU1 in HCC malignancy, we silenced circRSU1 in both HLF and Huh7 cells using antisense oligonucleotides (ASOs) of circRSU1. They were designed to target the BSJ site of circRSU1 so that only circRSU1 was significantly silenced without affecting the linear RSU1 mRNA (Figure 2A). Meanwhile, in both Huh7 and HLF cells, circRSU1 silencing significantly suppressed HCC spheroid formation assessed with two different methods (Figure 2B,C) and HCC colony formation (Figure 2D).

The effect of circRSU1 on tumor malignancy was further validated in vivo. Huh7 cells transfected with either ASO‐control or ASO‐circRSU1s were injected subcutaneously into nude mice for a tumor formation assay. Silencing circRSU1 significantly delayed tumor onset and inhibited tumor growth (Figure 2E; Figure S2A).

To further investigate the relationship between circRSU1 and HCC stemness, flow cytometry was performed to assess the proportion of cells positive for known hepatic CSC markers, including CD24, CD44, CD133, and EpCAM. Overexpression of circRSU1 specifically increased the proportion of CD24^positive^ cells, while silencing circRSU1 decreased CD24^positive^ population in both Huh7 and HLF cell lines (Figure 2F). CD24 is a well‐established hepatic CSC marker [7, 22]. Consistently, the isolated CD24^positive^ Huh7 cells exhibited a significantly enhanced spheroid formation and tumorigenesis capacity (Figure S2B,C), and silencing CD24 also reduced the spheroid formation (Figure S2D). These results collectively indicated that silencing circRSU1 significantly suppresses HCC malignancy both in vitro and in vivo and reduces CD24^positive^ CSC cell population.

### 2.3 CircRSU1 Interacted with RNA‐Binding Protein hnRNPA1, but did not Interact with miRNAs or Encode Proteins in HCC Cells

CircRNAs typically function through three ways, interacting with RNA binding proteins, acting as a miRNA sponge, or potentially encoding a protein. However, circRSU1 did not seem to encode protein (Figure S3A–C). ORFfinder prediction (https://www.ncbi.nlm.nih.gov/orffinder/) [23] showed that the circRSU1 sequence contained only an incomplete open reading frame (ORF) lacking a stop codon. IRESite prediction (http://www.iresite.org/) [24] showed that the potential internal ribosome entry site (IRES) sequence within circRSU1 scored low. The pBiCis dual‐luciferase reporter assay further demonstrated that the predicted IRES of circRSU1 only exhibited a very low protein translational potential (Figure S3C). The classical HIF‐1α IRES sequence was used as a positive control. Given these results, it was unlikely that circRSU1 encoded proteins.

**FIGURE 3:**
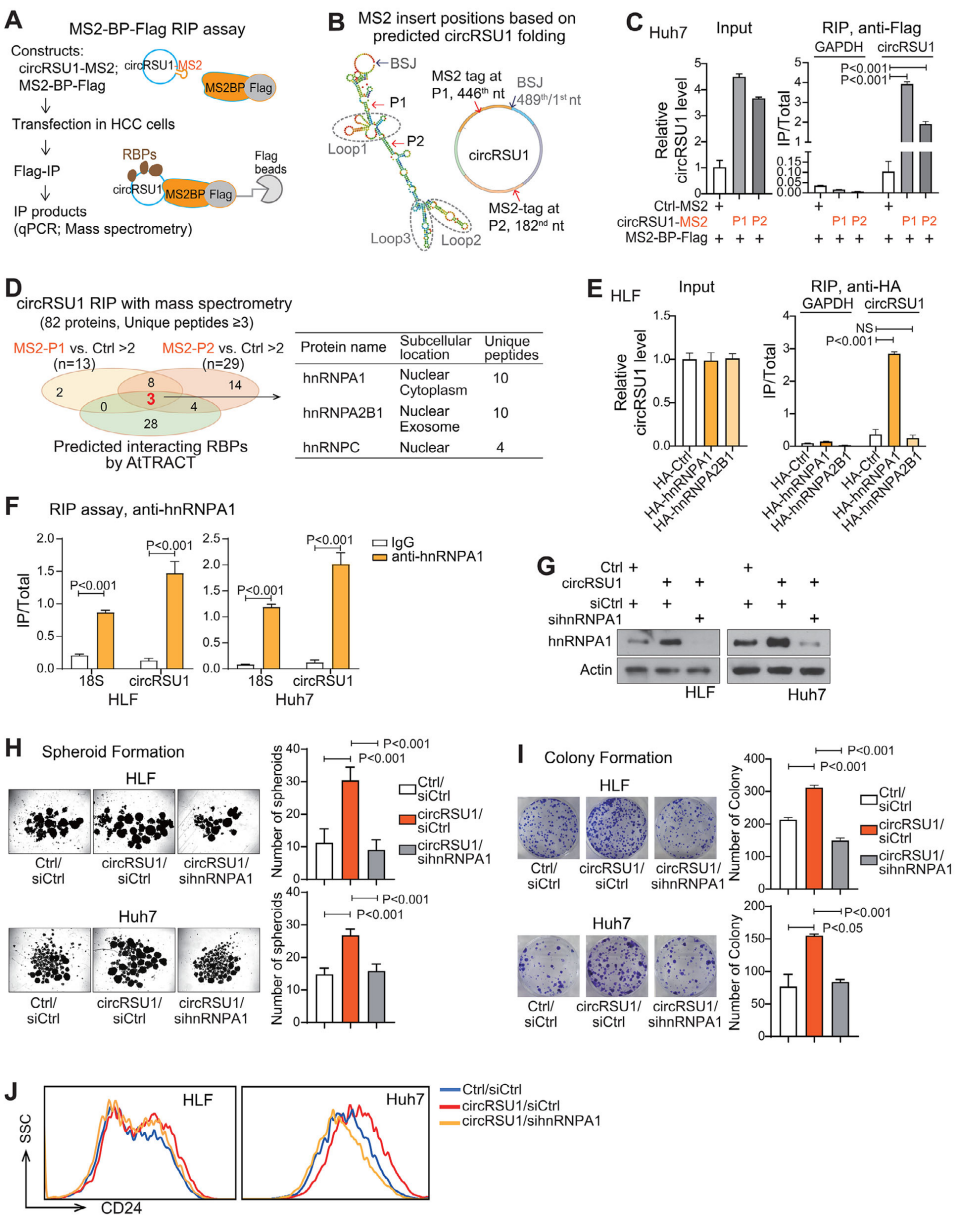
The RBP hnRNPA1 is a key downstream target of circRSU1. (A) Schematic flow chart of the MS2‐RIP assay‐based screening strategy to identify circRSU1‐interacting molecules. (B) Predicted secondary RNA structure of circRSU1 and the designed MS2 sequence insertion site on circRSU1. (C) Enrichment of circRSU1 in the MS2‐Flag RIP assay using anti‐Flag beads in Huh7 cells. Cells were co‐transfected with circRSU1‐MS2 and MS2‐Flag. GAPDH was used as the control RNA. (D) The mass spectrometry results of RIP products from Huh7 cells. Venn diagram analysis showed the identified RBPs from two independent RIP‐MS results with unique peptides≥3 and fold changes >2 in comparison with ctrl‐MS2 RIP‐MS results. Among them, three RBPs were predicted to interact with circRSU1 in the AtTRACT database. (E) The RIP assay was performed using anti‐HA beads in HLF cells transfected with HA‐hnRNPA1 or HA‐hnRNPA2B1 and the enrichment of circRSU1 was assessed by PCR. GAPDH was used as the control RNA. (F) The endogenous RIP assay was performed using hnRNPA1 antibody in Huh7 and HLF cells and the enrichment of circRSU1 was assessed by PCR. 18S RNA was used as the control RNA. (G) Western blot analysis of hnRNPA1 protein in Huh7 and HLF cells transfected with circRSU1 with or without silencing hnRNPA1. (H) Spheroid formation with low‐attachment plates in Huh7 and HLF cells transfected with circRSU1 with or without silencing hnRNPA1. (I) Colony formation assay in Huh7 and HLF cells transfected with circRSU1 with or without silencing hnRNPA1. (J) Flow cytometric analysis was performed to assess the proportion of CD24^positive^ cells in Huh7 and HLF cells transfected with circRSU1 with or without silencing hnRNPA1. (C, E, F, H, I) Statistical analysis was performed using Student's t‐test.

The CircInteractome online tool (https://circinteractome.nia.nih.gov/) [25] revealed 11 miRNAs with circRSU1 interacting site (one for each) (Figure S3A,D). The ATtRACT database (https://attract.cnic.es/) [26] showed 35 RNA‐binding proteins (RBPs) with more than three binding sites on circRSU1 (Figure S3A,E). To experimentally identify molecules interacting with circRSU1, we performed MS2‐RNA immunoprecipitation (RIP) assay utilizing the high affinity and specificity between the bacteriophage MS2 protein and its MS2 stem‐loop RNA structure (Figure 3A). Two circRSU1 vectors were constructed via incorporating MS2 stem‐loops in the sparse secondary RNA structure region of circRSU1 to minimize the possible disruption to circRSU1's structure and function. They were circRSU1‐MS2‐P1 with the MS2 stem‐loop inserted at the 446th nt and circRSU1‐MS2‐P2 at the 182nd nt (Figure 3B). In Huh7 cells, circRSU1‐MS2 and MS2‐Flag fusion protein were co‐transfected and the RIP assay was performed with an anti‐Flag antibody. Both circRSU1‐MS2 vectors efficiently overexpressed circRSU1 at levels comparable to the unmodified circRSU1 vector. The MS2‐Flag RIP assay significantly enriched circRSU1 compared to ctrl‐MS2 vector (Figure 3C) and to the unmodified circRSU1 vector (Figure S4).

With the RIP products, RT‐PCR was performed to examine the interacting miRNAs and mass spectrometry (MS) was performed to detect the interacting RBPs. Based on the RIP RT‐PCR results, circRSU1 less likely interacted with miRNAs in HCC cells. Majority of the 11 predicted miRNAs had low abundance or even were under detectable in HCC tumor tissues from HCC Cohort 2 (Figure S5A). Further, PCR detection with RIP products confirmed that circRSU1 did not enrich the three relatively high abundance miRNAs in two HCC cell lines (Figure S5B,C).

RIP‐MS results showed that 11 proteins were commonly identified from RIP‐MS assays with circRSU1‐MS2‐P1 and circRSU1‐MS2‐P2, with unique peptides ≥ 3 and fold changes > 2 compared to the ctrl‐MS2 group (Figure 3D). Among these, three RBPs (hnRNPA1, hnRNPA2B1, and hnRNPC) were predicted to interact with circRSU1 according to the ATtRACT database. Of these three RBPs, hnRNPA1 was likely a key RBP interacting with circRSU1 due to its high number of unique peptides (n = 10) detected in RIP‐MS and its cytoplasmic localization (Figure 3D), which was further confirmed by a series of RIP assays. An exogenous RIP assay showed that HA‐hnRNPA1 noticeably interacted with circRSU1 whereas HA‐hnRNPA2B1 did not, indicating the specificity of the interaction between hnRNPA1 and circRSU1 (Figure 3E). Consistent data were obtained when an endogenous RIP assay was performed with anti‐hnRNPA1 antibody (Figure 3F). Collectively, circRSU1 strongly interacts with the RBP hnRNPA1, which might be important for circRSU1's role in promoting HCC malignancy features.

### 2.4 hnRNPA1 was a Key Downstream Target of circRSU1 in Promoting HCC Malignancy and Stemness Features

hnRNPA1, heterogeneous nuclear ribonucleoprotein A1, is a well‐characterized RBP. It was overexpressed in numerous cancers including HCC, and increased some tumor malignancy phenotypes [27, 28]. In line with this, silencing hnRNPA1 significantly reduced HCC colony formation in both HLF and Huh7 cells (Figure S6A,B). Moreover, silencing hnRNPA1 also significantly reduced the spheroid formation, one of the stemness features (Figure S6C), and reduced the CD24^positive^ CSC population, while leaving other CSC marker positive populations (EpCAM, CD133, and CD44) mainly unaffected (Figure S6D). Consistent with these findings, in HCC patients from HCC cohorts 2–3, hnRNPA1 exhibited a significantly higher level in CD24^positive^ HCC tumor tissues compared to CD24^negative^ HCC tissues, when we defined the CD24^positive^ and CD24^negative^ HCCs based on the tertile cut‐off of CD24 (Figure S6E). These data indicated that hnRNPA1 was important in promoting HCC malignancy and stemness features.

To determine whether hnRNPA1 was the key downstream mediator of circRSU1‐induced malignancy and stemness features, we silenced hnRNPA1 in HCC cells with overexpressed circRSU1 (Figure 3G). In both HLF and Huh7 cells, circRSU1 significantly enhanced spheroid formation and colony formation, which however were ceased by silencing hnRNPA1 (Figure 3H,I). Moreover, flow cytometric analysis demonstrated that silencing hnRNPA1 diminished the enrichment of the CD24^positive^ population mediated by circRSU1 in both Huh7 and HLF HCC cells (Figure 3J). Collectively, these results indicated hnRNPA1 as a key downstream effector for circRSU1‐induced HCC malignancy and stemness features.

### 2.5 Two RNA Motifs of circRSU1 were Important for Interacting with hnRNPA1

**FIGURE 4:**
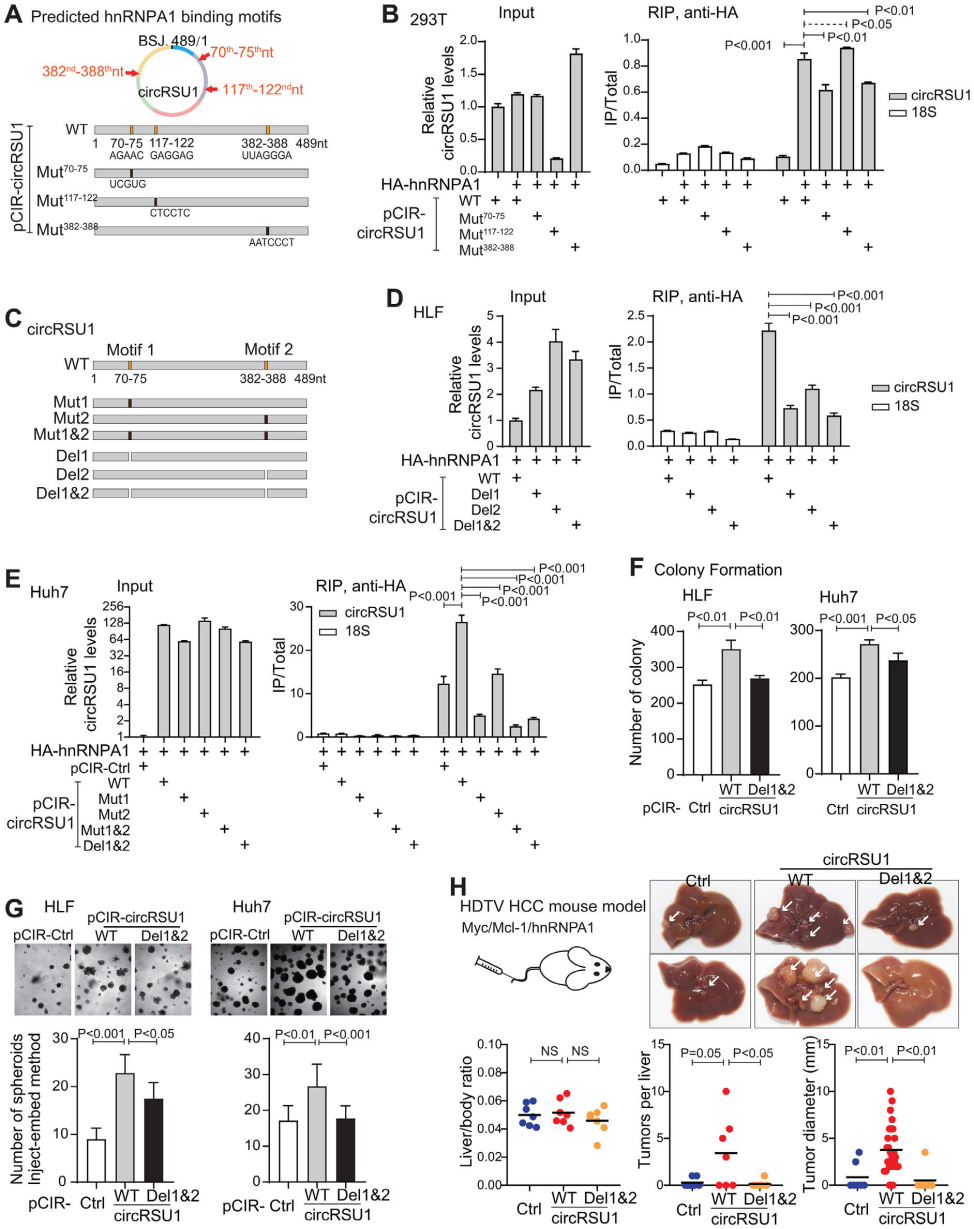
The RNA motifs of circRSU1 binding with hnRNPA1. (A) Schematic representation of the three predicted hnRNPA1‐binding motifs (70th–75th nt, 117th–122nd nt, and 382nd–388th nt) within the circRSU1 sequence, and a set of circRSU1 mutation vectors. (B) The exogenous RIP assay using anti‐HA beads in 293T transfected with HA‐hnRNPA1 and a series of circRSU1 vectors. The enrichment of circRSU1 was assessed by PCR and 18S RNA was used as the control RNA. (C) Schematic of hnRNPA1‐binding motifs’ mutation and deletion within the circRSU1. (D) The exogenous RIP assay using anti‐HA beads in HLF cells transfected with HA‐hnRNPA1 and a series of circRSU1 vectors. The enrichment of circRSU1 was assessed by PCR and 18S RNA was used as the control RNA. (E) The exogenous RIP assay using anti‐HA beads in Huh7 cells transfected with HA‐hnRNPA1 and a series of circRSU1 vectors. The enrichment of circRSU1 was assessed by PCR and 18S RNA was used as the control RNA. (F,G) Colony formation (F) and spheroid formation using the inject‐embed method (G) in Huh7 and HLF cells transfected with the wild‐type circRSU1 or circRSU1‐Del1&2. (H) HCC tumor formation in Myc/Mcl1‐induced HCC orthotopic mouse model with the presence of hnRNPA1 and different forms of circRSU1. Representative images were shown. Liver vs. body ratio, tumor numbers per liver, and tumor diameter were quantified and compared. Seven mice per group were used. (B, D–H) Student's t‐test was used for statistical analysis.

We then investigated the interaction motifs of circRSU1 and hnRNPA1. Based on the ATtRACT tool and the circRSU1 secondary structure, three potential hnRNPA1‐binding motifs on circRSU1 were predicted, i.e., 70th–75th nt, 117th–122nd nt, and 382nd–388th nt. The corresponding mutations were then constructed (Figure 4A). In 293T cells, these mutants were transfected together with HA‐hnRNPA1 and the RIP assay was performed with anti‐HA beads. The results revealed that the interaction between hnRNPA1 and circRSU1 was significantly reduced when motif 70th–75th nt or 382nd–388th nt was mutated, but not when motif 117th–122nd nt was mutated (Figure 4B). Consistent data were obtained in HLF cells (Figure S7). Thus, the 70th–75th nt region was termed as circRSU1 motif 1, and the 382nd–388th nt region as circRSU1 motif 2, which were important for circRSU1 to interact with hnRNPA1.

Further, additional circRSU1 mutations were constructed including a double mutation of motifs 1 and 2 (Mut1&2), motif 1 or 2 deletion (Del1, Del 2), and a double deletion of motifs 1 and 2 (Del1&2) (Figure 4C). Consistently in HLF cells, all these mutations reduced the interaction of circRSU1 with hnRNPA1 (Figure 4D). Comparable data were obtained in Huh7 cells, where both motif mutants and deletions of circRSU1 significantly reduced the interaction between circRSU1 and hnRNPA1 (Figure 4E).

Both in vitro and in vivo functional assays also showed that role of circRSU1 in promoting HCC malignancy and stemness features relied on the two hnRNAPA1‐interacting motifs. In vitro, the colony formation and spheroid formation were significantly reduced when these two motifs of circRSU1 were deleted in both HCC cell lines (Figure 4F,G). In vivo, an orthotopic HCC mouse model driven by Myc/Mcl1 was used. Due to the limited conservation of circRSU1 in its hnRNPA1 binding site between human and mouse (Figure S8A), we included human circRSU1 and hnRNPA1 for testing. Significantly, human circRSU1 promoted HCC tumor formation under the existence of human hnRNPA1, shown by the increased tumor numbers and tumor sizes, while circRSU1‐Del1&2 did not (Figure 4H). Within the limited tissues from this model, mouse Cd24 showed a higher level in circRSU1‐WT group than the control group and circRSU1‐Del1&2 group, supporting the circRSU1/CD24 signal axis in vivo (Figure S8B–D). Together, circRSU1 motifs 1 and 2 were important for its interaction with hnRNPA1 and its role in promoting HCC malignancy features.

### 2.6 Two RNA Binding Domains of hnRNPA1 were Important for Interacting with circRSU1 and circRSU1‐Mediated HCC Malignancy Features

**FIGURE 5:**
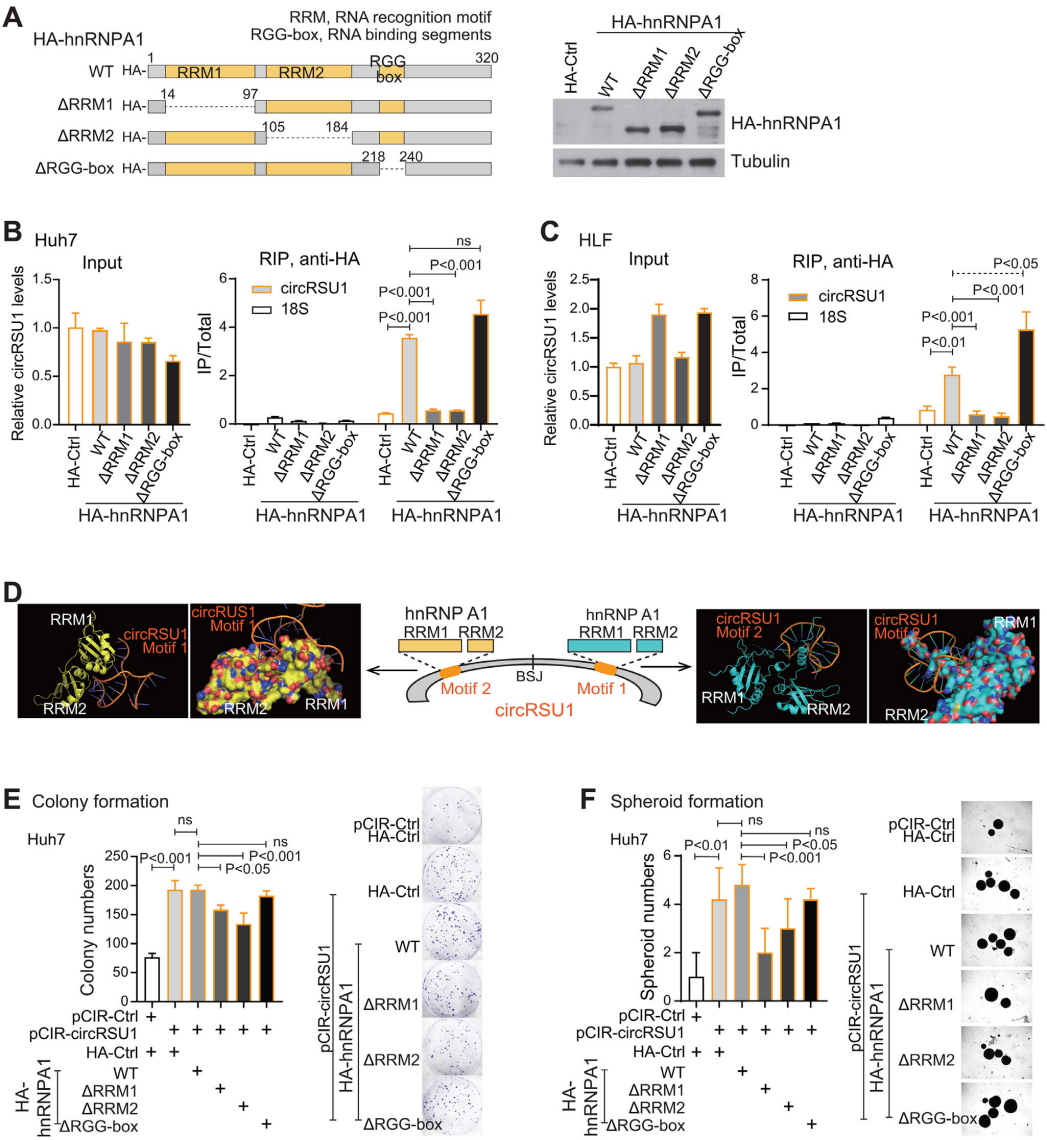
The protein domains of hnRNPA1 interacting with circRSU1. (A) Schematic representation of the functional domains of hnRNPA1 and various HA‐hnRNPA1 truncation constructs. Their expression efficiency was measured. (B,C) Enrichment of circRSU1 was assessed following the RIP assay with anti‐HA beads in Huh7 (B) and HLF (C) cells transfected with various HA‐hnRNPA1 vectors. (D) The docking of circRSU1 motifs and hnRNPA1 RBD domains using the HDOCK tool. (E,F) Colony formation (E) and spheroid formation with low‐attachment plates (F) of Huh7 cells with the co‐expressed circRSU1 and various HA‐hnRNPA1 vectors. (B, C, E, F) Statistical analysis was performed using Student's t‐test.

Next, we explored the protein domains of hnRNPA1 that interacted with circRSU1. Three domains of hnRNPA1 are responsible for its RNA recognition, i.e., RNA recognition motif 1 (RRM1, 14th–97th aa), RRM2 (105th–184th aa), and the Arg‐Gly‐Gly containing (RGG) Box (218th–240th aa). The corresponding hnRNPA1 deletions of each RNA recognition domain were constructed (Figure 5A). In HLF and Huh7 cells, RIP assays with anti‐HA‐hnRNPA1 revealed that the enrichment of circRSU1 by hnRNPA1 was largely reduced when either RRM1 or RRM2 domain was deleted, but not when RGG domain was removed (Figure 5B,C). Thus, the binding of hnRNPA1 to circRSU1 primarily depended on its RRM1 and RRM2 domains.

We then modeled the interaction between the hnRNPA1 protein and circRSU1 (Figure 5D) with the HDOCK online tool [29, 30]. The 3D RNA structure of circRSU1 motif 1 and motif 2 regions (approximately 28 nt) was predicted with the ROSIE online tool (https://rosie.graylab.jhu.edu/) [29] and the protein structures of hnRNPA1 RRM1 and RRM2 domains were retrieved from the RCSB Protein Data Bank [31]. As shown in Figure 5D, the interacting model consistently indicated that either motif 1 or motif 2 of circRSU1 interacted with protein structures formed with two hnRNPA1's RRM domains. Meanwhile, the spatial distance between two circRSU1 motifs likely allowed each motif to bind a separate hnRNPA1 protein, rather than a single hnRNPA1 interacting with two circRSU1 motifs simultaneously (Figure S9A).

Further, the oncogenic effects of circRSU1 in HCC also relied on the two RRM domains of hnRNPA1. In Huh7 cells, when either RRM1 or RRM2 domain of hnRNPA1 was deleted, the increased colony formation mediated by circRSU1 was significantly reduced (Figure 5E). As a negative control, the deletion of RGG domain of hnRNPA1 did not affect the enhanced colony formation mediated by circRSU1. Comparable data were obtained when spheroid formation was performed in Huh7 cells (Figure 5F). Meanwhile, consistent data of colony formation and spheroid formation were also obtained in HLF cells (Figure S9B,C). Together, hnRNPA1 interacted with circRSU1 through its RRM1 and RRM2 domains, which were important for circRSU1 promoting HCC malignancy features.

### 2.7 CircRSU1 Enhanced the hnRNPA1 Protein Level via Increasing its Protein Stability

**FIGURE 6:**
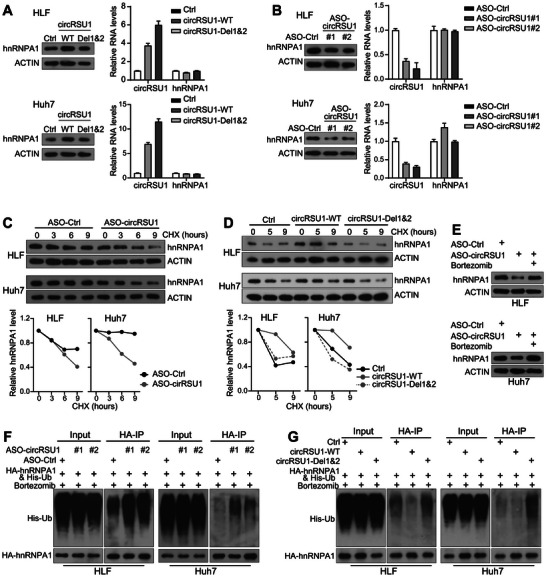
circRSU1 increases the hnRNPA1 protein stability. (A) RNA and protein levels of circRSU1 and hnRNPA1 were analyzed in Huh7 and HLF cell lines transfected with circRSU1‐WT or circRSU1‐Del1&2 vectors. (B) RNA and protein levels of circRSU1 and hnRNPA1 in Huh7 and HLF cell lines transfected with ASO‐Ctrl or ASO‐circRSU1. (C) HnRNPA1 protein level in Huh7 and HLF cells transfected with ASO‐Ctrl or ASO‐circRSU1 and treated with CHX at the indicated times. (D) HnRNPA1 protein level in Huh7 and HLF cells transfected with circRSU1‐WT or circRSU1‐Del1&2 vectors and treated with CHX at the indicated times. (E) hnRNPA1 protein level in Huh7 and HLF cells transfected with ASO‐Ctrl or ASO‐circRSU1, and treated with or without bortezomib. (F) The immunoprecipitation was performed to examine the polyubiquitination levels of hnRNPA1 in Huh7 and HLF cells co‐transfected with HA‐hnRNPA1 and His‐Ub plasmids, along with ASO‐Ctrl or ASO‐circRSU1. (G) The immunoprecipitation was performed to examine the polyubiquitination levels of hnRNPA1 in Huh7 and HLF cells co‐transfected with HA‐hnRNPA1 and His‐Ub plasmids, along with control, circRSU1‐WT, or circRSU1‐Del1&2 vectors.

Overexpression of circRSU1 in HCC cell lines noticeably increased hnRNPA1 protein level (Figure 3G). This circRSU1‐mediated increase in hnRNPA1 protein level was further confirmed in both Huh7 and HLF cells (Figure 6A). As a control, overexpressed circRSU1 did not alter the mRNA level of hnRNPA1. Meanwhile, when the circRSU1 motifs 1 and 2 were removed (circRSU1‐Del1&2), it no longer increased hnRNPA1 protein level. Consistently, silencing circRSU1 significantly decreased hnRNPA1 protein level in both Huh7 and HLF cells, while having no effect on reducing hnRNPA1 mRNA level (Figure 6B). These data indicate that circRSU1 increases hnRNPA1 protein at the post‐transcriptional level.

The cycloheximide (CHX) assay further showed that silencing circRSU1 reduced hnRNPA1 protein stability in both HCC cell lines (Figure 6C). Comparably, overexpressed wild‐type circRSU1 increased hnRNPA1 protein stability, whereas circRSU1‐Del1&2 did not (Figure 6D). HnRNPA1 has been reported to degrade through ubiquitin‐proteasome degradation system with ubiquitination at Lys8 mediated by an E3 ZFP91 [32]. As shown in Figure 6E, the proteasome inhibitor bortezomib restored the hnRNPA1 protein level reduced by circRSU1 silencing. The ubiquitination assay of hnRNPA1 further confirmed the role of circRSU1 in reducing the proteasome mediated hnRNPA1 degradation. In Huh7 and HLF cell lines, both circRSU1 ASO#1 and #2 increased polyubiquitination of hnRNPA1 (Figure 6F). Similarly, overexpression of wild‐type circRSU1 reduced hnRNPA1 polyubiquitination, while the circRSU1‐Del1&2 did not (Figure 6G). Comparable data were also obtained in 293T cells (Figure S10A). However, when Lys8 of hnRNPA1 was mutated to arginine (K8R), silencing circRSU1 still significantly decreased hnRNPA1^K8R^ protein level (Figure S10B). Additionally, no interaction between ZFP91 and hnRNPA1 was detected in HCC cells (Figure S10C). These data indicated that the interaction of circRSU1 with hnRNPA1 disturbed the ubiquitination of hnRNPA1 but not likely at its Lys8 (a known ubiquitination site), consequently enhancing hnRNPA1 protein stability and its protein level.

### 2.8 hnRNPA1 Enhanced HIF‐1α Protein Translation via Binding to the Internal Ribosome Entry Site (IRES) Region of HIF1A Gene

**FIGURE 7:**
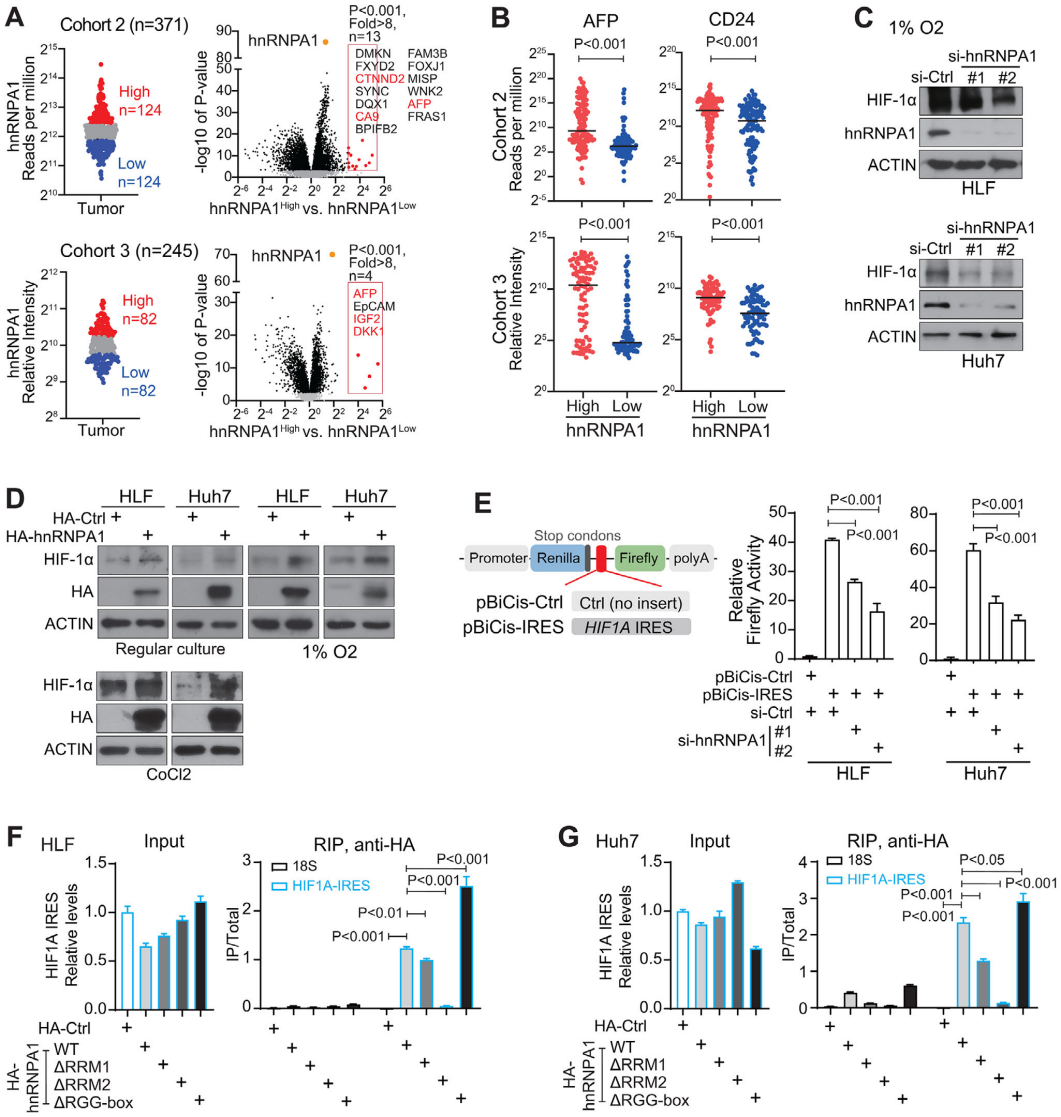
hnRNPA1 increases HIF‐1α protein translation via binding to its IRES region in HCC. (A) Differentially expressed genes between hnRNPA1^high^ and hnRNPA1^low^ groups were analyzed in HCC cohorts 2–3. Genes with significant high expression in hnRNPA1^high^ group were typed on the images. Genes in red color refer to the known HIF‐1α targets. (B) AFP and CD24 expression in hnRNPA1^high^ and hnRNPA1^low^ groups of cohorts 2 and 3. (C) HIF‐1α protein level in HLF and Huh7 cells transfected with si‐Ctrl or si‐hnRNPA1s under hypoxic condition (1% O_2_). (D) Western blot analysis of HIF‐1α protein level in HLF and Huh7 transfected with HA‐hnRNPA1. Cells were cultured under normoxia (21% O_2_), hypoxia (1% O_2_), or cobalt chloride treatment (Huh7 100 µm; HLF 200 µm) for 24 h post‐transfection. (E) Schematic presentation of pBiCis‐Ctrl and pBiCis‐IRES dual‐luciferase reporter constructs and the dual‐luciferase assay in HLF and Huh7 cells transfected with pBiCis‐IRES and si‐Ctrl or si‐hnRNPA1. (F,G) RIP assay with anti‐HA beads in HLF and Huh7 cells transfected with various HA‐hnRNPA1 vectors. RT‐qPCR was performed to measure the enrichment of *HIF1A* IRES RNA fragments, with 18S serving as the control RNA. (A, B, E‐G) Statistical analysis was performed using Student's t‐test.

To investigate how the circRSU1/hnRNPA1 axis promoted HCC malignance and stemness phenotypes, the transcriptomic data from two HCC cohorts were analyzed. In both cohorts, patients were stratified into high and low hnRNPA1 groups based on the tertile cut‐off of hnRNPA1 in HCC tumors in each cohort. Class comparison between hnRNPA1^high^ and hnRNPA1^low^ groups revealed that several HIF‐1α target genes were significantly highly expressed in hnRNPA1^high^ groups compared to hnRNPA1^low^ groups (P<0.001 and fold change>8) including AFP (Figure 7A). CD24 was also a known HIF‐1α target gene [33]. Consistently, both AFP and CD24 presented significantly higher levels in hnRNPA1^high^ HCC tumors compared to hnRNPA1^low^ tumors (Figure 7B). Consistent data were obtained in HCC Cohort 4 with proteomic data from 101 HCC tumor tissues [34] (Figure S11). Among the top 11 proteins with significantly higher levels in hnRNPA1^high^ HCCs vs. hnRNPA1^low^ HCCs (P < 0.05, fold‐change > 16), two proteins exhibit a close relationship with HIF‐1α. They are ENO2 as a known target of HIF‐1α [35] and IGF2BP3 that increases HIF‐1α level by binding to its mRNA [36]. Moreover, gene set enrichment analysis (GSEA) was performed with genes showing significantly differential expression (P<0.001) between hnRNPA1^high^ and hnRNPA1^low^ groups in cohorts 2–3. The analysis in both cohorts consistently revealed that gene sets associated with HCC stemness, aggressive proliferation, and poor patient survival were significantly enriched in the hnRNPA1^high^ ‐HCC group (Figure S12). AFP and CD24 are both HIF‐1α targets and hepatic CSC markers [7, 8, 22]. In this case, hnRNPA1 was likely involved in activating HIF‐1α pathway, which in turn mediated the expansion of CD24^positive^ cell population and enhanced HCC malignant phenotypes. Due to the short half‐life and low protein level of HIF‐1α under normoxia condition (21% O_2_), we examined the effect of silencing hnRNPA1 on HIF‐1α under hypoxic condition (1% O_2_). As shown in Figure 7C, silencing endogenous hnRNPA1 led to a significant reduction of HIF‐1α protein in both HLF and Huh7 HCC cells. Similarly, overexpressing hnRNPA1 further increased HIF‐1α protein level under hypoxic condition, as well as under normoxia condition (Figure 7D). Comparable data were also noticed even when cobalt chloride (CoCl_2_) was used to stabilize HIF‐1α (Figure 7D).

HnRNPA1 was reported to improve IRES‐mediated translation of several proteins [37]. It is known that the *HIF1A* IRES sequence allows efficient HIF‐1α protein translation. We then tested whether the hnRNPA1 enhanced the IRES‐mediated HIF‐1α protein translation in HCC cells, leading to an increased HIF‐1α protein level. A pBiCis‐IRES of HIF1A reporter system was used, in which the Firefly reporter was transcribed along with Renilla, but its translation relied on the *HIF1A* IRES [22]. Dual‐luciferase assay revealed that silencing hnRNPA1 significantly reduced Firefly luciferase activity mediated by *HIF1A* IRES (Figure 7E) in both HLF and Huh7 cells. Thus, hnRNPA1 increased HIF‐1α protein level via enhancing *HIF1A* IRES‐mediated translation of HIF‐1α protein.

Next, we evaluated the interaction between hnRNPA1 and the *HIF1A* IRES, and mapped the binding domains of hnRNPA1 responsible for this interaction (Figure 7F,G). First, a strong interaction between hnRNPA1 and the *HIF1A* IRES was noticed when the wild‐type hnRNPA1 was present. However, when the RRM2 domain of hnRNPA1 was deleted, such an interaction was largely reduced or even diminished. In contrast, deletion of hnRNPA1 RRM1 domain mildly reduced the interaction between hnRNPA1 and *HIF1A* IRES, and deletion of hnRNPA1 RGG‐box did not reduce this interaction at all. Consistent data were obtained in both HLF and Huh7 cells (Figure 7F,G). Thus, hnRNPA1 bound to the *HIF1A* IRES predominantly through its RRM2 domain, which promoted the HIF‐1α translation, leading to an increased HIF‐1α protein level in HCC cells.

### 2.9 CircRSU1 Further Enhanced the Interaction of hnRNPA1 and *HIF1A* IRES, Boosting the hnRNPA1/HIF1‐1α/CD24^positive^ Cell Axis

**FIGURE 8:**
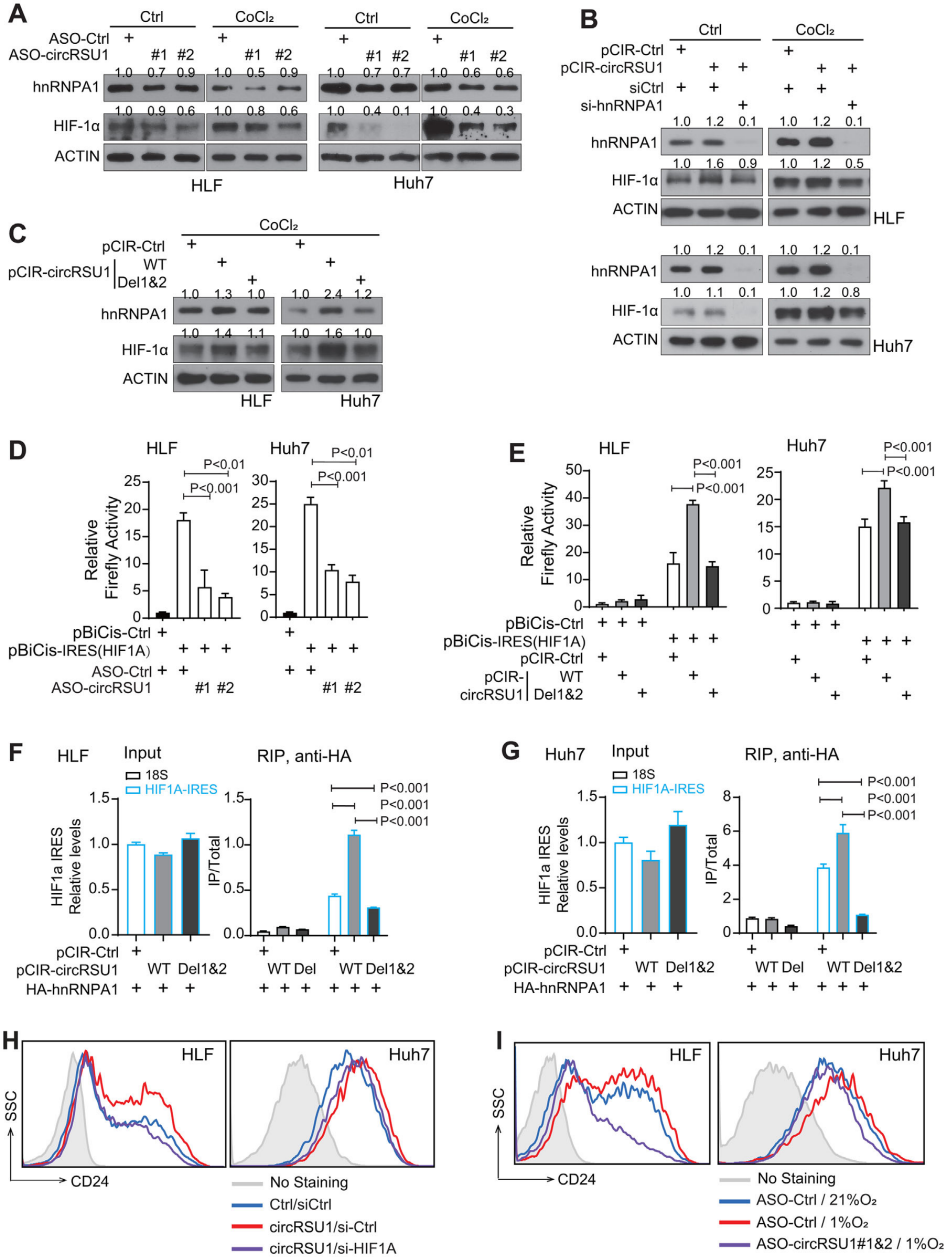
circRSU1 regulates HIF‐1α protein translation via hnRNPA1, enhancing CD24^positive^ subpopulation. (A) HIF‐1α protein level in HLF and Huh7 cells transfected with ASO‐control or ASO‐circRSU1s under control or CoCl_2_ treatment. (B) HIF‐1α protein level in HLF and Huh7 cells with the altered levels of circRSU1 and hnRNPA1 under control or CoCl_2_ treatment. (C) HIF‐1α protein level in HLF and Huh7 cells transfected with various circRSU1 vectors with CoCl_2_ treatment. (A–C) The quantitative protein levels of hnRNPA1 and HIF‐1α were shown. (D) Dual‐luciferase assay was performed to assess the effect on *HIF1A* IRES activity in HLF and Huh7 cells transfected with ASO‐control or ASO‐circRSU1s. (E) Dual‐luciferase assay was performed to assess the effect on *HIF1A* IRES activity in HLF and Huh7 cells transfected with control, circRSU1‐WT, or circRSU1‐Del1&2 vectors. (F,G) RIP assay with anti‐HA beads in HLF and Huh7 cells transfected with HA‐hnRNPA1 and various circRSU1 vectors. RT‐qPCR was performed to measure the enrichment of *HIF1A* IRES RNA fragments, with 18S serving as the control RNA. (H) Flow cytometry with anti‐CD24 antibody in HLF and Huh7 cells with the altered levels of circRSU1 and HIF‐1α. (I) Flow cytometry with anti‐CD24 antibody in HLF and Huh7 cells transfected with ASO‐Ctrl or ASO‐circRSU1 under normoxia or hypoxia conditions. (D–G) Statistical analysis was performed using Student's t‐test.

We further investigated the role of circRSU1 in regulating HIF‐1α. In both HLF and Huh7 cells, silencing circRSU1 noticeably reduced HIF‐1α protein level under either control condition or hypoxia‐mimicking conditions (CoCl_2_ treatment) (Figure 8A). Comparably, circRSU1 overexpression significantly increased HIF‐1α in both cell lines and both conditions (Figure 8B). Moreover, silencing hnRNPA1 in circRSU1 overexpression group significantly reduced the circRSU1‐mediated increase of HIF‐1α protein level in both cell lines (Figure 8B). Deletion of the hnRNPA1‐interacting motifs in circRSU1 (circRSU1‐Del1&2) abolished its ability on enhancing either hnRNPA1 or HIF‐1α protein level (Figure 8C). As a positive control, wild‐type circRSU1 did so. Together, these results demonstrate that circRSU1 enhances HIF‐1α level through interacting with hnRNPA1.

Consistent with hnRNPA1 enhancing *HIF1A* IRES activity, circRSU1 also increased the *HIF1A* IRES‐mediated protein translation. In both HLF and Huh7 cells, the dual‐luciferase assay with the pBiCis‐IRES (*HIF1A*) reporter system revealed that silencing circRSU1 significantly inhibited *HIF1A* IRES‐mediated luciferase activity (Figure 8D, P<0.001). Vice versa, overexpressing circRSU1 significantly induced the *HIF1A* IRES‐mediated luciferase activity. Meanwhile, such an effect was significantly reduced when the hnRNPA1‐interacting motifs in circRSU1 were deleted, i.e., the circRSU1‐Del1&2 group (Figure 8E). In addition, circRSU1 RIP assay with the MS2 system in both HCC cell lines revealed that circRSU1 did not enrich the *HIF1A* IRES fragment, ruling out the possibility of a direct RNA‐RNA interaction between circRSU1 and *HIF1A* IRES (Figure S13). Together, these results indicated that circRSU1 enhanced the *HIF1A* IRES‐mediated protein translation through interacting with hnRNPA1. As a negative control, circRSU1 did not appear to increase HIF‐1α protein stability (Figure S14A). MG132, a proteosome inhibitor, did not fully rescue the HIF‐1α reduction mediated by circRSU1 silencing (Figure S14B) and circRSU1 could further increase HIF‐1α level under hypoxia (Figure S14C).

HnRNPA1 bound to the *HIF1A* IRES via its RRM2 domain, which was also used to interact with circRSU1. We then tested whether the overexpressed circRSU1 disturbed the binding of hnRNPA1 with *HIF1A* IRES. RIP assays with anti‐hnRNPA1 showed that overexpression of circRSU1 did not weaken the interaction between hnRNPA1 and *HIF1A* IRES but even significantly enhanced their interaction (Figure 8F,G). In contrast, circRSU1‐Del1&2, lacking the hnRNPA1 interaction motifs, failed to promote and even significantly inhibited the hnRNPA1‐ *HIF1A* IRES interaction (Figure 8F,G). Data were consistent across both HLF and Huh7 cells. Thus, the circRSU1‐hnRNPA1 interaction was essential for hnRNPA1 binding to *HIF1A* IRES, which led to an enhanced HIF‐1α protein translation. Collectively, these findings demonstrated that circRSU1 enhanced HIF‐1α protein translation likely through both mechanisms, i.e., increasing hnRNPA1 protein level, and enhancing the binding of hnRNPA1 on *HIF1A* IRES.

Consistently with these findings, circRSU1 increased CD24^positive^ cell population via the hnRNPA1/HIF‐1α axis. Hypoxia induced CD24 level and silencing HIF1A significantly reduced CD24 (Figure S15A,B). Flow cytometry analysis showed that circRSU1 increased CD24^positive^ population, which was reduced when HIF‐1α was silenced in both HLF and Huh7 cells (Figure 8H; Figure S15C). Meanwhile, silencing circRSU1 via ASOs reduced CD24^positive^ population in HCC cells, which was enhanced when HIF‐1α was induced under hypoxia condition (Figure 8I; Figure S15D). Comparable data were also obtained for CD24 mRNA level (Figure S15E,F). Collectively, circRSU1 promotes HCC stemness and malignancy features by activating the hnRNPA1/HIF‐1α/CD24 signaling axis.

## 3 Discussion

HCC exhibits significant tumor heterogeneity features, with subpopulations of cells possessing “stem cell‐like” self‐renewal and differentiation potential that can initiate tumor occurrence. It has been shown that these subpopulations contribute to chemotherapeutic resistance, recurrence, and metastasis in HCC [9, 38, 39]. It is thus crucial of identifying the key players in regulating malignant progression, particularly in maintaining the tumor stem cell populations (e.g., CD24^positive^ CSCs). Here in this study, we revealed circRSU1 as one key regulator of CD24^positive^ cell population and HCC malignancy features via activating the hnRNPA1/HIF‐1α/CD24 signaling axis.

Via the expression profiling and function screening, we identified circRSU1 as a highly abundant circular RNA in HCC tumor with important roles in promoting cancer stemness. A group of validation assays and mechanistic studies established circRSU1 as a key regulator in enhancing HCC malignancy features and CD24^positive^ subpopulation via the hnRNPA1/HIF‐1α/CD24 signaling axis. First, circRSU1 showed a significantly higher level in FFPE tumor tissues compared to adjacent non‐tumor tissues from HCC patients. In HCC cell lines, silencing circRSU1 significantly reduced HCC cell colony formation, spheroid formation, tumorigenesis, and CD24^positive^ cell population. Second, circRSU1 interacted with the RNA binding protein hnRNPA1, with their interaction domains mapped. Third, the interaction of hnRNPA1 and circRSU1 enhanced hnRNPA1 protein stability. Fourth, not only the enhanced level of hnRNPA1 but also the circRSU1/hnRNPA1 interaction increased the interaction of hnRNPA1 with *HIF1A* IRES, boosting HIF‐1α protein translation, a key player of HCC stemness and malignancy features. Consequently, circRSU1 promoted HCC malignancy features and CD24^positive^ subpopulation via the hnRNPA1/HIF‐1α axis, contributing to HCC stemness and heterogeneity.

Recently, three studies implicated circRSU1 in osteoarthritis, retinal vascular dysfunction under diabetic conditions, and lung microvascular endothelial cell damage [40, 41, 42]. Combined with our findings, it suggests that circRSU1 plays diverse roles across multiple diseases. Given its high abundance in HCC FFPE tissues and the detectable level in non‐tumor liver FFPE tissues, it would be interesting to further investigate circRSU1 expression in other tissues, mechanisms of its altered expression, and roles of the circRSU1/hnRNPA1/HIF‐1α/CD24 axis in the above diseases and other cancers.

As a classical RNA‐binding protein, hnRNPA1 regulates various RNA‐related processes, such as transcription, splicing, translation, mRNA stability, and transport. Meanwhile, hnRNPA1 could promote tumorigenesis and progression, with elevated expression in highly metastatic liver cancer cell lines and tumor tissues [43]. It enhanced the expression level of CD44v6 [43], promoted the splicing of the pyruvate kinase PKM2 [44], and inhibited the cyclin‐dependent kinase inhibitor P16INK4a [45], contributing to HCC progression. Given that circRSU1 increased the hnRNPA1 protein level, these downstream targets of hnRNPA1, except HIF‐1α, might also contribute to circRSU1‐mediated HCC malignancy features. Since the CD24^positive^ cell population was specifically increased in circRSU1 overexpression cells, we thus mainly focused on how the circRSU1/hnRNPA1 axis driven this phenotype. In this case, we discovered the interaction of hnRNPA1 and *HIF1A* IRES, by which hnRNPA1 increased the HIF‐1α protein translation, leading to an increased CD24^positive^ cell population. Meanwhile, circRSU1 enhanced the interaction of hnRNPA1 and *HIF1A* IRES and the hnRNPA1 protein level, further boosting the HIF‐1α translation. This is consistent with reports that hnRNPA1 can promote the IRES‐dependent translation of other proteins [46, 47, 48]. In this case, it was also likely that circRSU1 acted as an IRES trans‐acting factor by interacting with hnRNPA1, potentially facilitating the translation of many other proteins. Further in‐depth studies on hnRNPA1, especially in CD24^positive^ HCC patients, might be informative to disclose the therapeutic potential of hnRNPA1 as a target in HCC.

CD24 is a highly glycosylated protein anchored to the cell membrane via glycosyl‐phosphatidylinositol (GPI). It has been revealed as a CSC marker in multiple cancers including HCC [7, 49]. Our previous research revealed the miR‐125b/HIF‐1α axis contributes to a significant and specific increase in the CD24^positive^ CSC population [22]. Together with this study, these results indicated the critical role of hypoxia and HIF‐1α regulation in enriching CD24^positive^ CSC population. Meanwhile, CD24 has also been shown as a “don't eat me” signal on tumor cells of ovarian and breast cancers, by interacting with the immune‐suppressive receptor Siglec‐10 on immune cells, thereby protecting tumor cells from phagocytosis [50, 51]. Whether CD24 also mediated immune evasion in HCC remained undiscovered. Thus, it seemed to be very important to continuously explore the role of hypoxic tumor microenvironment in regulating CD24^positive^ cells and their possible tumor immune evasion in HCC. All these information might collectively pave the way to target the CD24^positive^ cells and discover its potential utilization in treating HCC patients.

Overall, we revealed circRSU1 as a key player in promoting HCC malignancy features and enriching CD24^positive^ CSC population via the axis of circRSU1/hnRNPA1/HIF‐1α. These findings not only provided the insight of circRSU1’ role in HCC malignancy and stemness but also demonstrated a specific regulatory axis of CD24^positive^ CSC population, contributing to HCC heterogeneity feature.

## 4 Material and Methods

### 4.1 Clinical Specimens and Databases

A total of four distinct HCC cohorts and three circRNA datasets were used in this study (Table S1). Cohort 1 comprised formalin‐fixed paraffin‐embedded (FFPE) tissues from 61 HCC patients of the Shandong Cancer Hospital and Institute, China. Cohort 2 included 371 HCC patients, with mRNA sequencing data available for 371 tumor tissues and 50 non‐tumor liver tissues, sourced from The Cancer Genome Atlas (TCGA, https://portal.gdc.cancer.gov). HCC Cohort 3 consisted of 245 HCC patients from China, with available transcriptomic data for paired tumor and adjacent non‐tumor tissues (GSE14520). HCC Cohort 4 included 101 HCC patients with proteomic data available in their tumor tissues, sourced from (https://www.nature.com/articles/s41586‐019‐0987‐8#MOESM1) [34]. The study was approved by the institutional review board at each study center.

In addition to these cohorts, three circRNA datasets were utilized to screen circRNA candidates with high abundance in HCC tumors. Dataset 1 included paired circRNA microarray data from tumor and paired non‐tumor tissues of seven HCC patients (GSE97322); circRNA Dataset 2 included circRNA microarray data from tumor and paired non‐tumor tissues of five HCC patients (GSE94508); and circRNA Dataset 3 included circRNA sequencing data of paired tumor and non‐tumor tissue data from five HCC patients, published in *Journal of Hepatology* [14].

### 4.2 Plasmids, Antisense Oligonucleotides (ASOs), and siRNAs

For overexpressing circRNAs, the pCircRNA (pCIR) vector was generated with the known Alu1 and Alu2 sequences, which could facilitate the cleavage and circularization of the fragment between them [52]. They were ligated together via a short linker containing XhoI and EcoRI restriction sites. The resulting fragment was then inserted into the HindIII/XbaI sites of the pcDNA3.0 vector. For the pCIR‐circRNA candidates, the full‐length sequence of the circRNA candidate was amplified by PCR and subsequently inserted into the linearized pCIR vector (digested with EcoRI/XhoI) using the ClonExpress MultiS One Step Cloning Kit (Vazyme, Cat# C113‐02) by homologous recombination. For the pCIR‐circRSU1‐MS2‐P1 and pCIR‐circRSU1‐MS2‐P2 vectors, the MS2 stem‐loop sequence (containing two tandem repeats) was synthesized and inserted into the linearized pCIR‐circRSU1 vector at the P1 (446 nt) and P2 (182 nt) sites by homologous recombination using the ClonExpress MultiS One Step Cloning Kit. For the construction of pCIR‐circRSU1‐Mut1, pCIR‐circRSU1‐Mut2, pCIR‐circRSU1‐Mut3, pCIR‐circRSU1‐Mut1&3, pCIR‐circRSU1‐Del1, pCIR‐circRSU1‐Del3, and pCIR‐circRSU1‐Del1&3 vectors, PCR amplification was used to generate the truncated or mutated sequences of circRSU1. These fragments were then inserted into the linearized pCIR vector by enzyme digestion of EcoRI/XhoI using the ClonExpress MultiS One Step Cloning Kit via homologous recombination. For the pBiCis circRSU1‐IRES vector, the predicted IRES sequence of circRSU1 was amplified by PCR, and subsequently inserted into the NtoI/SacI sites of the pBiCis‐Ctrl vector via restriction enzyme digestion and ligation. For the construction of pcDNA3.0‐HA‐hnRNPA1, pcDNA3.0‐HA‐hnRNPA2B1, pcDNA3.0‐HA‐hnRNPA1‐Δ14‐97, pcDNA3.0‐HA‐hnRNPA1‐Δ105‐184, pcDNA3.0‐HA‐hnRNPA1‐Δ218‐240, and pcDNA3.0‐HA‐hnRNPA1‐K8R vectors, the full‐length sequence of hnRNPA1, the full‐length sequence of hnRNPA2B1, or the truncated sequences of hnRNPA1 were amplified by PCR. The resulting fragments were then inserted into the EcoRI/XhoI sites of the pcDNA3.0‐HA vector via restriction enzyme digestion and ligation. Vectors MS2‐Flag, His‐MYC‐Ub, pBiCis‐Ctrl, and pBiCis‐HIF1A‐IRES were constructed and used as previously described [22, 53, 54]. The sequence information for these primers is listed in Table S2.

ASOs targeting circRSU1 and control ASOs were designed and purchased from GenePharma Co., Ltd., Shanghai, China. SiRNAs of hnRNPA1, CD24, and HIF‐1α, as well as control siRNAs, were also obtained from GenePharma Co., Ltd. Detailed sequence information of these ASOs and siRNAs are listed in Table S3.

### 4.3 Cell Culture and Treatment

Human embryonic kidney cell line 293T and human HCC cell lines Huh7, Huh1, HLF, and HLE were cultured in Dulbecco's Modified Eagle Medium (DMEM) supplemented with 10% fetal bovine serum, 100 U/mL penicillin‐streptomycin, and 1% L‐glutamine. All cell lines were maintained in a humidified atmosphere of 5% CO_2_ at 37°C. Hypoxic environment was used via culturing cells in a hypoxia incubator (Biospherix) with the setting of 1% O_2_, 5% CO_2_. The Huh7, Huh1, HLF, and HLE cell lines were obtained from the Japanese Collection of Research Bioresources Cell Bank (JCRB), and the 293T cell line was sourced from the American Type Culture Collection (ATCC).

Plasmid transfections were performed using Lipofectamine 2000 Reagent (Invitrogen, Cat#11668019), and transfections of siRNAs and ASOs were conducted using Rfect siRNA Transfection Reagent (BIOTRAN, Cat#11011). Bortezomib (Sigma–Aldrich, Cat# B‐0057) and MG132 (MCE, HY13259) were dissolved in dimethyl sulfoxide (DMSO) and used to inhibit proteasome‐mediated degradation. Cycloheximide (CHX) (Selleck, Cat#S7418) was used in protein synthesis inhibition experiments. HCC cells were treated with CHX at a concentration of 20 µg/mL and analyzed at various time points. Cobalt chloride (CoCl_2_) (Sigma–Aldrich, Cat#15862) was used to chemically induce a hypoxia condition via stabilizing HIF‐1α. To detect HIF‐1α stability, cells were treated with CoCl_2_ for 36 h before exposure to CHX for the indicated time.

### 4.4 Mouse Studies

Animal experiments were approved by the Experimental Animal Committee of Zhejiang University and were conducted in accordance with the guidelines for animal welfare (ZJU20200014). BALB/c nude mice and ICR mice were purchased from Shanghai SLAC Laboratory Animal Co., Ltd. The mice were housed in the Zhejiang University Laboratory Animal Center under specific pathogen‐free conditions, maintained in laminar‐flow cabinets at room temperature with a 24‐hour light‐dark cycle.

For the tumorigenicity assay with circRSU1 silencing, 1×10^6^ Huh7 cells transfected with ASO‐Ctrl, ASO‐circRSU1#1, or ASO‐circRSU1#2 were suspended in 100 µl of PBS and Matrigel (Corning, Cat# 354284) (1:1) and subcutaneously implanted into the flanks of 4‐week‐old male BALB/c nude mice. Seven subcutaneous implantation sites were used per group. For the tumorigenicity assay with CD24^positive^ and CD24^negative^ Huh7 cells, they were first sorted and then suspended in PBS and Matrigel (1:1). 20 000 cells were subcutaneously implanted into the flanks of 4‐week‐old male BALB/c nude mice per site. Eight sites were used for CD24^positive^ group and four sites for CD24^negative^ group. Tumor formation was monitored twice weekly, and tumor volume was calculated using the following formula: Volume = 0.5 × Width^2^ × Length.

For the oncogene‐induced orthotopic HCC tumorigenicity assay, a Sleeping Beauty (SB) transposon system and six‐week‐old wild‐type ICR female mice were used as we did before via hydrodynamic tail vein injection [55]. The combination of pT3‐EF1α‐Myc/pT3‐EF1α‐Mcl1/pT3‐EF1α‐hnRNPA1(5/5/10 µg) along with pCMV/SB (1.2 µg) was introduced to induce HCC formation. pT3‐EF1α‐circRSU1 and pT3‐EF1α‐circRSU1‐Del1&2 vectors (20 µg for each) were used to test their function in regulating HCC formation. For each injection, the combined plasmids were diluted in 2 mL saline (0.9% NaCl), filtered through a 0.22 µm filter, and injected into the lateral tail vein of mice in 5 to 7 s at a volume equivalent to 1/10 of the mouse's body volume.

### 4.5 Structure Prediction of circRSU1, Spatial Folding Prediction of circRSU1 Motifs, and Docking Analysis of circRSU1 and hnRNPA1

To predict the secondary structure of circRSU1, its 489‐nucleotide full‐length sequence was retrieved from the circBank database (https://www.circbank.cn/#/home) and analyzed using the RNAfold web server (http://rna.tbi.univie.ac.at/cgi‐bin/RNAWebSuite/RNAfold.cgi). For spatial folding prediction of circRSU1, the sequence was submitted to the RNAComposer web server (https://rnacomposer.cs.put.poznan.pl/), which supports RNA structure modeling for sequences up to 500 nts. To further investigate the structural features of the circRSU1's functional regions involved in protein interaction, we predicted the spatial folding of the identified key circRSU1 motifs. This was done using FARFAR (Fragment Assembly of RNA with Full‐Atom Refinement) analysis via the ROSIE web server (https://rosie.graylab.jhu.edu/rna_denovo/submit). Specifically, the nucleotide sequences of each circRSU1 motif were individually submitted to ROSIE to obtain their predicted spatial folding models.

To explore how circRSU1 and hnRNPA1 interact, molecular docking analysis was performed using their predicted spatial folding models. The hnRNPA1 protein structure, which includes two RNA recognition motifs (RRM1 and RRM2) obtained from the RCSB Protein Data Bank. The predicted spatial folding models of circRSU1 motif 1 and motif 2 served as the receptor molecules, while the two RRM domains of hnRNPA1 were set as the ligand molecules. Docking was conducted using the HDOCK web server (http://hdock.phys.hust.edu.cn), yielding structural models that illustrate potential interactions between hnRNPA1 and circRSU1 motif.

### 4.6 RNA Extraction, RNase R Digestion, and Quantitative Real‐Time PCR

Total RNA was extracted using TRIzol RNA Isolation Reagent (Invitrogen) according to the manufacturer's protocol. For the RNase R digestion assay, RNA samples were treated with RNase R enzyme (Epicentre, Cat# 30250–1) for the indicated time following the manufacturer's instructions. cDNA was synthesized from 1 µg of RNA using the PrimeScriptTM RT Reagent Kit (TaKaRa, Cat# RR047) for detecting mRNA expression. For high‐throughput miRNA detection, cDNA was synthesized from 1 µg of RNA using the Mir‐X miRNA First‐Strand Synthesis Kit (TaKaRa, Cat# 638315). Quantitative reverse transcription polymerase chain reaction (qRT‐PCR) was performed using TB Green Premix Ex Taq II (TaKaRa, Cat# RR420). GAPDH and 18S rRNA were used as the reference genes for the detected mRNAs. U6 was used as the reference gene for the detected miRNAs. All primer sequences for PCR detection are listed in Table S4.

### 4.7 Cytoplasmic and Nuclear RNA Fractionation

Cytoplasmic and nuclear RNA fractions of HCC cell lines were extracted using the PARIS Kit (Invitrogen, Cat# AM1921) according to the manufacturer's protocol. Briefly, a total of 1 × 10^6^ cells were pelleted, washed, and resuspended in 300 µl of ice‐cold Cell Fractionation Buffer. Ten minutes after the incubation, centrifuge was performed at 500 g for 5 min at 4°C, and the supernatant was collected as the cytoplasmic fraction. The nuclear pellet was washed with 300 µl of ice‐cold Cell Fractionation Buffer and centrifuged at 500 g for 5 min at 4°C. The nuclear pellet was then resuspended in 300 µl of ice‐cold Cell Disruption Buffer, and lysed by vigorous vortex. For both the cytoplasmic and nuclear fractions, 300 µl of Lysis/Binding Solution was added, followed by mixing with 100% ethanol. The samples were then washed with Wash Solution using spin columns, and RNAs were eluted into collection tubes, respectively. RNA concentration was measured and stored for further analysis.

### 4.8 RNA Immunoprecipitation (RIP) Assay

For the RIP assay, cells from one 10 cm culture dishes were lysed in 1 mL of RIP buffer (25 mm Tris‐HCl pH 7.5, 150 mm KCl, 0.5 mm DTT, 0.5% NP‐40, 2 µL RiboLock RNase Inhibitor (Invitrogen, Cat# CAEO0382)) prepared with DEPC‐treated water, on ice for 30 min. The lysate was then centrifuged at 12 000 rpm for 20 min and the supernatant was collected. A 20 µL aliquot of the supernatant was collected as the Input sample and RNA was extracted using 1 mL of TRIzol. The remaining supernatant was incubated with the Pierce anti‐HA magnetic beads (Thermo Scientific, Cat# 88836) or protein A/G beads (Thermo Scientific, Cat#88802) with the presence of indicated antibodies at 4°C for 8 h. The beads were washed three times with NT2 buffer (50 mm Tris‐HCl pH 7.4, 150 mm NaCl, 1 mm MgCl_2_, 0.05% NP‐40) prepared in DEPC‐treated water, and RNA samples were subsequently collected using TRIzol for qPCR analysis.

### 4.9 Colony Formation Assay, Spheroid Formation Assay

For the colony formation assay, Huh7 or HLF (1000 cells/well) cells were seeded into 6 cm culture dishes and incubated for 12 days. Colonies were then fixed with methanol, stained with crystal violet, and counted. For the spheroid formation assay with the low‐attachment plates, Huh7 or HLF cells (1000 cells/well, or the indicated number) were seeded into the ultra‐low attachment 24‐well plate (Corning, Cat# 3473) and cultured for 10–12 days. Spheroids were subsequently counted under a microscope. For the spheroid formation with inject‐embed method, a 1:1 mixture of cell culture medium and Matrigel (Corning, Cat# 354284) was placed into the 96‐well culture plate with 50 µL per well. One hour after placing, the semi‐solid matrix was formed and Huh7 or HLF cells were gently injected into the matrix. An additional 50 µL of complete culture medium was carefully overlaid on the semi‐solid matrix. Spheroids were counted under a microscope. 1000 Huh7 cells/well were used and data were collected at 8 days after seeding. 2000 HLF cells/well were used and data were collected at 10 days after seeding.

### 4.10 Flow Cytometry Analysis and Cell Sorting

Cultured cells were harvested by trypsinization and washed with 1×PBS, followed by resuspension in 1×PBS containing 0.5% fetal bovine serum. The cells were then incubated with APC‐conjugated antibodies on ice for 30 min in the dark. The antibodies were anti‐CD24 antibody (Miltenyi Biotec, Cat#32D12), anti‐CD44 antibody (Miltenyi Biotec, Cat#DB105), anti‐CD133 antibody (Miltenyi Biotec, Cat#AC133), and anti‐EpCAM antibody (Miltenyi Biotec, Cat#HEA‐125). For incubation, 1 µl of anti‐CD24 antibody and 2 µl of anti‐CD44, EpCAM, and CD133 antibody per 1 × 10^6^ cells was used. Data were collected with FACSCalibur flow cytometer (BD Biosciences) and analyzed with FlowJo software.

For fluorescence‐activated cell sorting (FACS) of CD24^positive^ and CD24^negative^ cells, Huh7 cells were incubated with anti‐CD24 antibody (Miltenyi Biotec, Cat#32D12) and then sorted using a BD FACSAria II flow cytometer (BD Bioscience). The top 10% of strongly stained cells were sorted as positive cells, and the bottom 10% of weakly stained cells were sorted as negative cells.

### 4.11 Protein Extraction, Western Blotting, and CHX Assay

Cells were lysed on ice for 30 min in IP buffer (1% NP40, 150 mm NaCl, 50 mm Tris pH 7.4, 10% glycerol). The cell lysates were separated by SDS‐PAGE Gel and transferred to PVDF membranes. The membranes were incubated with the indicated primary antibodies, followed by incubation with horseradish peroxidase‐conjugated secondary antibodies for enhanced chemiluminescence detection. The antibodies included anti‐hnRNPA1 (Abclonal, Cat#A11564), anti‐HIF‐1α (Proteintech, Cat#20960‐1‐AP), anti‐HA (Cell Signaling Technology, Cat# 3724), anti‐His (Cell Signaling Technology, Cat# 2365), anti‐ZFP91 (Thermo Scientific, Cat#PA5‐41199), anti‐Actin (ABclonal, Cat# AC026), HRP‐linked anti‐rabbit IgG antibody (Jackson Immuno Research, lot.129736), and HRP‐linked anti‐mouse IgG antibody (Jackson Immuno Research, lot.129457). To detect protein stability, cells were treated with 20 µg/ml CHX (Selleck, Cat#S7418) and collected at the indicated time points for western blot assay.

### 4.12 MS2‐Flag Immunoprecipitation (IP) and Mass Spectrometry

For the MS2‐Flag immunoprecipitation assay, Huh7 cells were cultured in 10 cm culture dishes and co‐transfected with the MS2‐Ctrl, MS2‐circRSU1‐P1, MS2‐circRSU1‐P2, and MS2‐Flag vectors. After 48 h of transfection, cells were washed twice with ice‐cold 1× PBS and lysed in IP buffer on ice for 30 min. The lysate was then centrifuged at 12 000 rpm for 20 min to separate the supernatant. The supernatant was incubated with anti‐Flag‐M2 magnetic beads (Sigma–Aldrich, Cat#M8823) at 4°C for 8 h. After washing, for RNA analysis, RNA was extracted using TRIzol and subjected to subsequent qPCR analysis. For protein analysis, the beads containing immunoprecipitated proteins were boiled in 1×SDS loading buffer, and then used for mass spectrometry analysis in our institute.

### 4.13 Dual‐Luciferase Assay

pBiCis dual‐luciferase reporter plasmid containing both the firefly luciferase gene and the Renilla luciferase gene was used. The firefly luciferase gene was regulated by the target IRES sequence, while the Renilla luciferase gene was driven by the CMV promoter, serving as a control for normalization. Luciferase activity was measured using the Dual‐Luciferase Reporter Assay System at 48 h post transfection. Firefly luciferase activity was normalized to Renilla luciferase activity.

### 4.14 Ubiquitination Assay

To detect ubiquitylation of hnRNPA1 in HLF, Huh7, and 293T cells, cells were co‐transfected with His‐MYC‐Ub, HA‐hnRNPA1 along with ASO‐circRSU1s or pCIR‐circRSU1 vectors. Cells were lysed in IP buffer. The collected cell lysates were incubated with the Pierce anti‐HA magnetic beads (Thermo Scientific, Cat# 88836) at 4°C for 6 h. The immunocomplexes were washed four times using IP buffer. After washing, the immunoprecipitated proteins were subjected to immunoblotting.

### 4.15 Statistical Analysis

Class comparison was used to compare the expression levels of RNAs or proteins between groups in different circRNA datasets and cohorts 2–4. Gene set enrichment analysis (GSEA) in the Molecular Signatures Database was conducted using GSEA version 4.2.2. Two‐way ANOVA and Student's t‐test were employed for statistical comparison between groups. Kaplan‐Meier survival analysis was utilized to compare mouse tumor occurrence across different groups using GraphPad Prism version 8.0 (San Diego, CA). P‐values are generated by the log‐rank test. All P‐values were two‐sided, and a *p*‐value of less than 0.05 was considered statistically significant.

## Author Contributions

J.J., S.X., D.W., and Y.Z. conceived and designed the study, with J.J. providing overall supervision. Methodology development was led by S.X., D.W., Y.Z., and H.Z., while data acquisition was carried out by S.X., D.W., Y.Z., J.P., H.Z., N.L., and J.Z., Data analysis and interpretation were performed by S.X., D.W., Y.Z., H.Z., and J.J., and the manuscript was written and revised by S.X., D.W., Y.Z., S.R., and J.J., with additional administrative and technical support provided by L.Z., J.J., X.F., M.X., A.L., and J.L.

## Funding

This work was supported by the ‘Leading Goose’ R&D Program of Zhejiang Province (2023C03052) (J.Ji), National Natural Science Foundation of China (82361148723; 82073055) (J. Ji), the China Postdoctoral Science Foundation (2025M782778) (J. Zhang), the Joint Research Program of Shaoxing University and Shaoxing Institute, Zhejiang University (2023LHLG001) (J. Ji), the Fundamental Research Funds for the Central Universities in China (J. Ji), the German Research Foundation (DFG) project (469332207 and 493697503) (S. Roessler) and the German Cancer Aid (Deutsche Krebshilfe) project (70113922) (S. Roessler).

## Ethics Statement

All mouse procedures were conducted under the guidelines and the institutional animal care protocol approved by the Experimental Animal Committee at Zhejiang University (ZJU20200014, J.Ji).

## Conflicts of Interest

The authors declare no conflicts of interest.

## Supporting information




**Supporting File 1**: advs74339‐sup‐0001‐SuppMat.docx.

**Supporting File 2**: advs74339‐sup‐0002‐Data.xlsx.

## Data Availability

Data are available in a public, open access repository. The mRNA sequencing data of Cohort 2 were available at TCGA portal (https://portal.gdc.cancer.gov). The mRNA profiling data of Cohort 3 were available at GEO datasets of NCBI (GSE14520,https://www.ncbi.nlm.nih.gov/geo/query/acc.cgi?acc=GSE14520). The proteomic data of Cohort 4 were available at the ‘Source Data Extended Data Figure 2’ from Dr. He (Nature 2019, https://www.nature.com/articles/s41586‐019‐0987‐8#MOESM1). CircRNA dataset 1 included circRNA microarray data were available at GEO datasets of NCBI (GSE97322, https://www.ncbi.nlm.nih.gov/geo/query/acc.cgi?acc=GSE97322). CircRNA dataset 2 included circRNA microarray data were available at GEO datasets of NCBI (GSE94508,https://www.ncbi.nlm.nih.gov/geo/query/acc.cgi?acc=GSE94508). CircRNA dataset 3 included circRNA sequencing data were obtained from Dr. Yu (https://www.sciencedirect.com/science/article/pii/S0168827818300552?via%3Dihub). All data relevant to the study are included in the article or uploaded as online supplemental information.

## References

[1] H. Sung , J. Ferlay , R. L. Siegel , et al., “Global Cancer Statistics 2020: GLOBOCAN Estimates of Incidence and Mortality Worldwide for 36 Cancers in 185 Countries,” CA: A Cancer Journal for Clinicians 71 (2021): 209–249. 33538338 10.3322/caac.21660

[2] H. Qiu , S. Cao , and R. Xu , “Cancer Incidence, Mortality, and Burden in China: A Time‐Trend Analysis and Comparison with the United States and United Kingdom Based on the Global Epidemiological Data Released in 2020,” Cancer Communications 41 (2021): 1037–1048, 10.1002/cac2.12197. 34288593 PMC8504144

[3] H. Rumgay , M. Arnold , J. Ferlay , et al., “Global Burden of Primary Liver Cancer in 2020 and Predictions to 2040,” Journal of Hepatology 77 (2022): 1598–1606, 10.1016/j.jhep.2022.08.021. 36208844 PMC9670241

[4] A. Forner , M. Reig , and J. Bruix , “Hepatocellular Carcinoma,” The Lancet 391 (2018): 1301–1314, 10.1016/S0140-6736(18)30010-2. 29307467

[5] X. R. Yang , Y. Xu , B. Yu , et al., “High Expression Levels of Putative Hepatic Stem/Progenitor Cell Biomarkers Related to Tumour Angiogenesis and Poor Prognosis of Hepatocellular Carcinoma,” Gut 59 (2010): 953–962, 10.1136/gut.2008.176271. 20442200

[6] Y. Gu , X. Wei , Y. Sun , et al., “miR‐192‐5p Silencing by Genetic Aberrations is a Key Event in Hepatocellular Carcinomas with Cancer Stem Cell Features,” Cancer Research 79 (2019): 941–953, 10.1158/0008-5472.CAN-18-1675. 30530815 PMC6397664

[7] T. K. Lee , A. Castilho , V. C. Cheung , K. H. Tang , S. Ma , and I. O. Ng , “CD24^+^ Liver Tumor‐Initiating Cells Drive Self‐renewal and Tumor Initiation through STAT3‐mediated NANOG Regulation,” Cell Stem Cell 9 (2011): 50–63. 21726833 10.1016/j.stem.2011.06.005

[8] Y. Z. Zhao , D. D. Wei , Y. T. Zhang , and J. F. Ji , “Panoramic View of microRNAs in Regulating Cancer Stem Cells,” Essays in Biochemistry 66 (2022): 345–348. 35996948 10.1042/EBC20220007

[9] J. Ji and X. W. Wang , “Clinical Implications of Cancer Stem Cell Biology in Hepatocellular Carcinoma,” Seminars in Oncology 39 (2012): 461–472, 10.1053/j.seminoncol.2012.05.011. 22846863 PMC3409471

[10] J. Ji , T. Yamashita , A. Budhu , et al., “Identification of microRNA‐181 by Genome‐Wide Screening as a Critical Player in EpCAM–Positive Hepatic Cancer Stem Cells†,” Hepatology 50 (2009): 472–480, 10.1002/hep.22989. 19585654 PMC2721019

[11] T. Yamashita , J. Ji , A. Budhu , et al., “EpCAM‐Positive Hepatocellular Carcinoma Cells Are Tumor‐Initiating Cells with Stem/Progenitor Cell Features,” Gastroenterology 136 (2009): 1012–1024, 10.1053/j.gastro.2008.12.004. 19150350 PMC2828822

[12] H. Suzuki and T. Tsukahara , “A View of Pre‐mRNA Splicing from RNase R Resistant RNAs,” International Journal of Molecular Sciences 15 (2014): 9331–9342, 10.3390/ijms15069331. 24865493 PMC4100097

[13] I. L. Patop , S. Wüst , and S. Kadener , “Past, Present, and Future of circRNAs,” The EMBO Journal 38 (2019): 100836, 10.15252/embj.2018100836. PMC6694216 31343080

[14] J. Yu , Q. G. Xu , Z. G. Wang , et al., “Circular RNA cSMARCA5 Inhibits Growth and Metastasis in Hepatocellular Carcinoma,” Journal of Hepatology 68 (2018): 1214–1227, 10.1016/j.jhep.2018.01.012. 29378234

[15] J. Xu , L. Ji , Y. Liang , et al., “CircRNA‐SORE Mediates Sorafenib Resistance in Hepatocellular Carcinoma by Stabilizing YBX1,” Signal Transduction and Targeted Therapy 5 (2020): 298, 10.1038/s41392-020-00375-5. 33361760 PMC7762756

[16] Y. Wei , X. Chen , C. Liang , et al., “A Noncoding Regulatory RNAs Network Driven by Circ‐CDYL Acts Specifically in the Early Stages Hepatocellular Carcinoma,” Hepatology 71 (2020): 130–147. 31148183 10.1002/hep.30795

[17] D. Han , J. Li , H. Wang , et al., “Circular RNA circMTO1 Acts as the Sponge of microRNA‐9 to Suppress Hepatocellular Carcinoma Progression,” Hepatology 66 (2017): 1151–1164, 10.1002/hep.29270. 28520103

[18] L. Fu , T. Yao , Q. Chen , X. Mo , Y. Hu , and J. Guo , “Screening Differential Circular RNA Expression Profiles Reveals hsa_circ_0004018 is Associated with Hepatocellular Carcinoma,” Oncotarget 8 (2017): 58405–58416, 10.18632/oncotarget.16881. 28938566 PMC5601662

[19] X. Gu , J. Zhang , Y. Ran , et al., “Circular RNA hsa_circ_101555 Promotes Hepatocellular Carcinoma Cell Proliferation and Migration by Sponging miR‐145‐5p and Regulating CDCA3 Expression,” Cell Death & Disease 12 (2021): 356, 10.1038/s41419-021-03626-7. 33824281 PMC8024300

[20] Q. Zheng , C. Bao , W. Guo , et al., “Circular RNA Profiling Reveals an Abundant circHIPK3 That Regulates Cell Growth by Sponging Multiple miRNAs,” Nature Communications 7 (2016): 11215, 10.1038/ncomms11215. PMC4823868 27050392

[21] H. Miao , F. Wu , Y. Li , et al., “MALAT1 modulates Alternative Splicing by Cooperating with the Splicing Factors PTBP1 and PSF,” Science advances 8 (2022): abq7289. 10.1126/sciadv.abq7289 PMC9788761 36563164

[22] X. Wei , L. Zhao , R. Ren , et al., “MiR‐125b Loss Activated HIF1α/pAKT Loop, Leading to Transarterial Chemoembolization Resistance in Hepatocellular Carcinoma,” Hepatology 73 (2021): 1381–1398, 10.1002/hep.31448. 32609900 PMC9258000

[23] M. Liu , Q. Wang , J. Shen , B. B. Yang , and X. M. Ding , “Circbank: A Comprehensive Database for circRNA with Standard Nomenclature,” RNA Biology 16 (2019): 899–905, 10.1080/15476286.2019.1600395. 31023147 PMC6546381

[24] P. Kolekar , A. Pataskar , U. Kulkarni‐Kale , J. Pal , and A. Kulkarni , “IRESPred: Web Server for Prediction of Cellular and Viral Internal Ribosome Entry Site (IRES),” Scientific Reports 6 (2016): 27436. 27264539 10.1038/srep27436 PMC4893748

[25] D. B. Dudekula , A. C. Panda , I. Grammatikakis , S. De , K. Abdelmohsen , and M. Gorospe , “CircInteractome: A Web Tool for Exploring Circular RNAs and Their Interacting Proteins and microRNAs,” RNA Biology 13 (2016): 34–42, 10.1080/15476286.2015.1128065. 26669964 PMC4829301

[26] G. Giudice , F. Sanchez‐Cabo , C. Torroja , and E. Lara‐Pezzi , “ATtRACT—A Database of RNA‐binding Proteins and Associated Motifs,” Database(Oxford) 2016 (2016): baw035. 27055826 10.1093/database/baw035 PMC4823821

[27] H. Yang , R. Zhu , X. Zhao , et al., “Sirtuin‐Mediated Deacetylation of hnRNP A1 Suppresses Glycolysis and Growth in Hepatocellular Carcinoma,” Oncogene 38 (2019): 4915–4931, 10.1038/s41388-019-0764-z. 30858544

[28] X. Gao , Z. Wan , M. Wei , et al., “Chronic Myelogenous Leukemia Cells Remodel the Bone Marrow Niche via Exosome‐mediated Transfer of miR‐320,” Theranostics 9 (2019): 5642. 31534508 10.7150/thno.34813 PMC6735391

[29] S. Lyskov , F. C. Chou , S. O. Conchuir , et al., “Serverification of Molecular Modeling Applications: The Rosetta Online Server That Includes Everyone (ROSIE),” PloS one 8 (2013): 63906. 10.1371/journal.pone.0063906 PMC3661552 23717507

[30] Y. Yan , H. Tao , J. He , and S. Y. Huang , “The HDOCK Server for Integrated Protein–Protein Docking,” Nature Protocols 15 (2020): 1829–1852, 10.1038/s41596-020-0312-x. 32269383

[31] S. K. Burley , C. Bhikadiya , C. Bi , et al., “RCSB Protein Data Bank (RCSB.org): Delivery of Experimentally‐determined PDB Structures alongside One Million Computed Structure Models of Proteins from Artificial Intelligence/Machine Learning,” Nucleic Acids Research 51 (2023): D488–D508, 10.1093/nar/gkac1077. 36420884 PMC9825554

[32] D. Chen , Y. Wang , R. Lu , et al., “E3 Ligase ZFP91 Inhibits Hepatocellular Carcinoma Metabolism Reprogramming by Regulating PKM Splicing,” Theranostics 10 (2020): 8558. 32754263 10.7150/thno.44873 PMC7392027

[33] S. Thomas , M. A. Harding , S. C. Smith , et al., “CD24 is an Effector of HIF‐1–Driven Primary Tumor Growth and Metastasis,” Cancer Research 72 (2012): 5600–5612, 10.1158/0008-5472.CAN-11-3666. 22926560 PMC3488144

[34] Y. Jiang , A. Sun , Y. Zhao , et al., “Proteomics Identifies New Therapeutic Targets of Early‐Stage Hepatocellular Carcinoma,” Nature 567 (2019): 257–261, 10.1038/s41586-019-0987-8. 30814741

[35] J. Shi , D. Miao , Q. Lv , D. Tan , Z. Xiong , and X. Zhang , “ENO2 as a Biomarker Regulating Energy Metabolism to Promote Tumor Progression in Clear Cell Renal Cell Carcinoma,” Biomedicines 11 (2023): 2499. 37760940 10.3390/biomedicines11092499 PMC10525605

[36] L. Jiang , Y. Li , Y. He , D. Wei , L. Yan , and H. Wen , “Knockdown of m6A Reader IGF2BP3 Inhibited Hypoxia‐Induced Cell Migration and Angiogenesis by Regulating Hypoxia Inducible Factor‐1α in Stomach Cancer,” Frontiers in Oncology 11 (2021): 711207, 10.3389/fonc.2021.711207. 34621671 PMC8490730

[37] G. Gao , S. Dhar , and M. T. Bedford , “PRMT5 Regulates IRES‐Dependent Translation via Methylation of hnRNP A1,” Nucleic Acids Research 45 (2017): 4359–4369. 28115626 10.1093/nar/gkw1367 PMC5416833

[38] C. Saygin , D. Matei , R. Majeti , O. Reizes , and J. D. Lathia , “Targeting Cancer Stemness in the Clinic: from Hype to Hope,” Cell Stem Cell 24 (2019): 25–40, 10.1016/j.stem.2018.11.017. 30595497

[39] Y. Gu , X. Zheng , and J. Ji , “Liver Cancer Stem Cells as a Hierarchical Society: Yes or no?,” Acta Biochimica et Biophysica Sinica 52 (2020): 723–735, 10.1093/abbs/gmaa050. 32490517

[40] Y. Yang , P. Shen , T. Yao , et al., “Novel Role of circRSU1 in the Progression of Osteoarthritis by Adjusting Oxidative Stress,” Theranostics 11 (2021): 1877–1900, 10.7150/thno.53307. 33408787 PMC7778608

[41] Y. Zhang , J. Hu , X. Qu , and K. Hu , “Circular RNA RSU1 Promotes Retinal Vascular Dysfunction by Regulating miR‐345‐3p/TAZ,” Communications Biology 6 (2023): 719, 10.1038/s42003-023-05064-x. 37443201 PMC10344963

[42] Y. Cheng , F. Wang , C. Guo , S. Yuan , J. Li , and Y. Zhang , “CircRSU1 Alleviates LPS‐Induced Human Pulmonary Microvascular Endothelial Cell Injury by Targeting miR‐1224‐5p/ITGA5 Axis,” General physiology and biophysics 43 (2024): 1–11, 10.4149/gpb_2023031. 38312030

[43] Z. J. Zhou , Z. Dai , S. L. Zhou , et al., “Overexpression of HnRNP A1 Promotes Tumor Invasion through Regulating CD44v6 and Indicates Poor Prognosis for Hepatocellular Carcinoma,” International Journal of Cancer 132 (2013): 1080–1089, 10.1002/ijc.27742. 22821376

[44] H. R. Christofk , M. G. Vander Heiden , M. H. Harris , et al., “The M2 Splice Isoform of Pyruvate Kinase is Important for Cancer Metabolism and Tumour Growth,” Nature 452 (2008): 230–233, 10.1038/nature06734. 18337823

[45] B. Zhao , X. Lv , X. Zhao , et al., “Tumor‐Promoting Actions of HNRNP A1 in HCC are Associated with Cell Cycle, Mitochondrial Dynamics, and Necroptosis,” International Journal of Molecular Sciences 23 (2022): 10209. 36142139 10.3390/ijms231810209 PMC9499416

[46] F. Damiano , S. Alemanno , G. V. Gnoni , and L. Siculella , “Translational Control of the Sterol‐regulatory Transcription Factor SREBP‐1 mRNA in Response to Serum Starvation or ER Stress is Mediated by an Internal Ribosome Entry Site,” Biochemical Journal 429 (2010): 603–612, 10.1042/BJ20091827. 20513236

[47] H. J. Kim , H. R. Lee , J. Y. Seo , et al., “Heterogeneous Nuclear Ribonucleoprotein A1 Regulates Rhythmic Synthesis of Mouse Nfil3 Protein via IRES‐Mediated Translation,” Scientific Reports 7 (2017): 42882, 10.1038/srep42882. 28220845 PMC5318856

[48] O. D. Jo , J. Martin , A. Bernath , J. Masri , A. Lichtenstein , and J. Gera , “Heterogeneous Nuclear ribonucleoprotein A1 Regulates Cyclin D1 and c‐myc Internal Ribosome Entry Site Function through Akt Signaling,” Journal of Biological Chemistry 283 (2008): 23274–23287, 10.1074/jbc.M801185200. 18562319 PMC2516998

[49] M. Fogel , J. Friederichs , Y. Zeller , et al., “CD24 is a Marker for Human Breast Carcinoma,” Cancer Letters 143 (1999): 87–94, 10.1016/S0304-3835(99)00195-0. 10465342

[50] A. A. Barkal , R. E. Brewer , M. Markovic , et al., “CD24 Signalling through Macrophage Siglec‐10 is a Target for Cancer Immunotherapy,” Nature 572 (2019): 392–396, 10.1038/s41586-019-1456-0. 31367043 PMC6697206

[51] G. Y. Chen , J. Tang , P. Zheng , and Y. Liu , “CD24 and Siglec‐10 Selectively Repress Tissue Damage–Induced Immune Responses,” Science 323 (2009): 1722–1725, 10.1126/science.1168988. 19264983 PMC2765686

[52] D. Liang and J. E. Wilusz , “Short Intronic Repeat Sequences Facilitate Circular RNA Production,” Genes & Development 28 (2014): 2233–2247, 10.1101/gad.251926.114. 25281217 PMC4201285

[53] L. Jiang , M. Xiao , Q. Q. Liao , et al., “High‐Sensitivity Profiling of SARS‐CoV‐2 Noncoding Region–Host Protein Interactome Reveals the Potential Regulatory Role of Negative‐Sense Viral RNA,” Msystems 8 (2023): e00135. 37314180 10.1128/msystems.00135-23 PMC10469612

[54] J. Liu , B. Yuan , J. Cao , et al., “AMBRA1 Promotes TGFβ Signaling via Nonproteolytic Polyubiquitylation of Smad4,” Cancer Research 81 (2021): 5007–5020, 10.1158/0008-5472.CAN-21-0431. 34362797

[55] F. Ji , J. Zhang , N. Liu , et al., “Blocking Hepatocarcinogenesis by a Cytochrome P450 Family Member with Female‐Preferential Expression,” Gut 71 (2022): 2313–2324, 10.1136/gutjnl-2021-326050. 34996827

