# Supplementary material for: CircRSU1 Activates the hnRNPA1/HIF‐1α/CD24 Signaling Axis, Promoting Stemness Features of Hepatocellular Carcinoma
Source: Adv Sci (Weinh). 2026 Feb 12;13(22):e22424. doi: 10.1002/advs.202522424 (PMC13088275; doi:10.1002/advs.202522424)
Supplement: Supplementary file 1 — Supporting File 1: advs74339‐sup‐0001‐SuppMat.docx. [file ADVS-13-e22424-s001.docx]

***Supplementary Information***

**circRSU1 Activates the hnRNPA1/HIF-1α/CD24 Signaling Axis, Promoting Stemness Features of Hepatocellular C**

Shuting Xue^1,2,3,†^, Danduo Wei^1,2,3,†^, Yongzhi Zhao^1,2,3,†^, Jinchang Pan^1,2,3^, Hanyuan Zhang^1,2,3^, Niya Liu^1,2,3^, Jianjuan Zhang^1,2,3^, Jiaxin Liu^1,2,3^, Jianping Jin^1,2,3^, Mu Xiao^1,2,3^, Xinhua Feng^1,2,3^, Aifu Lin^3,4^, Lei Zhao^5^, Stephanie Roessler^6^, Junfang Ji^1,2,3,*^.

**Supplementary Tables, n=4 (Pages S2-S6)**

Table S1. Summary of datasets and clinical specimens used in this study.

Table S2. Primers for plasmids construction used in this study.

Table S3. Sequence of ASOs and siRNAs used in this study.

Table S4. PCR primers used in this study.

**Supplementary Figures, n=15 (Pages S7-S21)**

Figure S1. Identification of circRNAs with high levels in tumors and abilities of promoting stemness features in HCC.

Figure S2. The cancer stem cell features of CD24^positive^ cells.

Figure S3. The potential of circRSU1 in coding proteins or working as miRNA sponges.

Figure S4. Validation of the MS2 system in circRSU1 enrichment.

Figure S5. miRNA expression and miRNA enrichment by circRSU1.

Figure S6. Silencing hnRNPA1 significantly suppresses the malignant phenotype of HCC.

Figure S7. Enrichment of circRSU1 in RIP assay using anti-HA beads.

Figure S8. circRSU1 and hnRNPA1 expression efficiency in orthotopic tumorigenesis model.

Figure S9. The predicted spatial folding of circRSU1 and phenotypes of HLF cells co-expressing circRSU1 and different truncated forms of HA-hnRNPA1.

Figure S10. The effect of circRSU1 on the ubiquitination of hnRNPA1.

Figure S11. Class comparisons of proteome between HCCs with high hnRNPA1 and HCCs with low hnRNPA1 protein level.

Figure S12. The top 20 enriched signatures in hnRNPA1^high^ group of HCC cohorts 2-3.

Figure S13. circRSU1 does not directly bind to *HIF1A* IRES.

Figure S14. circRSU1 does not enhance HIF-1α protein stability.

Figure S15. The effects of *HIF1A* knockdown and hypoxic condition on CD24 and HIF-1α.

**Supplementary Tables**

**Table S1 Summary of datasets and clinical specimens used in this study**

| **Cohorts**  **/Datasets** | **Source** | **HCC cases** | **Datasets or Specimens** |
| --- | --- | --- | --- |
| HCC Cohort 1 | Shandong Cancer Hospital, China | 61 | Stored RNAs from FFPE tissues |
| HCC Cohort 2 | TCGA | 371 | mRNA sequencing data (371 tumor tissues; 50 non-tumor tissues) |
| HCC Cohort 3 | GEO datasets (GSE14520) | 245 | Transcriptomic data of the paired tumor and adjacent non-tumor tissues |
| HCC Cohort 4 | *Nature* | 101 | Proteomics data of tumor tissues |
| circRNA Dataset 1 | GEO datasets (GSE97322) | 7 | Paired circRNA microarray data from tumor and non-tumor tissues |
| circRNA Dataset 2 | GEO datasets (GSE94508) | 5 | Paired circRNA microarray data from tumor and non-tumor tissues |
| circRNA Dataset 3 | *Journal of Hepatology* | 5 | Paired circRNA sequencing data from tumor and non-tumor tissues |

**Table S2. Primers for plasmids construction used in this study**

| **Vector name** | **Primer sequence** |
| --- | --- |
| pCIR | **F:** ACCCAAGCTTAAAGTGCTGAGATTACAGGCGTGAG |
|  | **R:** AGAATTATCTAGAGTGCTGGGATTACAGGTGTGAG |
| circRSU1-Mut1 | **F1:** TTTTTTTATACTTCAGTTACCTTATCCCATATCACACAACTGGTC |
|  | **R1:** GAGCACCTCCAAATTCTTCACACGAGCGATGTTCGGTGGCACCATTG |
|  | **F2:** ATGGTGCCACCGAACATCGCTCGTGTGAAGAATTTGGAGGTGCTCAAC TT |
|  | **R2:** TTTCCTTGCTTCTTACCTAGTTCTGGGGGCAGAACG |
| circRSU1-Mut2 | **F1:** TTTTTTTATACTTCAGTTACCTTATCCCATATCACACAACTGGTC |
|  | **R1:** CTACTGATCTGTGTGGGCAGGAGGAGGATTTGGTTATTAAAAAAGTTGAGCACC TCCAA |
|  | **F2:** AACTTTTTTAATAACCAAATCCTCCTCCTGCCCACACAGATCAGTAGCC |
|  | **R2:** TTTCCTTGCTTCTTACCTAGTTCTGGGGGCAGAACG |
| circRSU1-Mut3 | **F1:** TTTTTTTATACTTCAGTTACCTTATCCCATATCACACAACTGGTC |
|  | **R1:** GGCAGCGAGATCAGGTCGTTAAGGGATTGGCTGAGTATCTGCAACTTTGTGAGC |
|  | **F2:** CAAAGTTGCAGATACTCAGCCAATCCCTTAACGACCTGATCTCGCTGCCTAAG |
|  | **R2:** TTTCCTTGCTTCTTACCTAGTTCTGGGGGCAGAACG |
| circRSU1-Mut1&3 | **F1:** TTTTTTTATACTTCAGTTACCTTATCCCATATCACACAACTGGTC |
|  | **R1:** GAGCACCTCCAAATTCTTCACACGAGCGATGTTCGGTGGCACCATTG |
|  | **F2:** ATGGTGCCACCGAACATCGCTCGTGTGAAGAATTTGGAGGTGCTCAACT T |
|  | **R2:** GGCAGCGAGATCAGGTCGTTAAGGGATTGGCTGAGTATCTGCAACTTTG  TGAGC |
|  | **F3:** CAAAGTTGCAGATACTCAGCCAATCCCTTAACGACCTGATCTCGCTGCCTAAG |
|  | **R3:** TTTCCTTGCTTCTTACCTAGTTCTGGGGGCAGAACG |
| circRSU1-Del1 | **F1:** TTTTTTTATACTTCAGTTACCTTATCCCATATCACACAACTGGTC |
|  | **R1:** GAGCACCTCCAAATTCTTCAGCGATGTTCGGTGGCACCATTG |
|  | **F2:** ATGGTGCCACCGAACATCGCTGAAGAATTTGGAGGTGCTCAACTT |
|  | **R2:** TTTCCTTGCTTCTTACCTAGTTCTGGGGGCAGAACG |
| circRSU1-Del3 | **F1:** TTTTTTTATACTTCAGTTACCTTATCCCATATCACACAACTGGTC |
|  | **R1:** GGCAGCGAGATCAGGTCGTTAGGCTGAGTATCTGCAACTTTGTGAGC |
|  | **F2:** CAAAGTTGCAGATACTCAGCCTAACGACCTGATCTCGCTGCCTAAG |
|  | **R2:** TTTCCTTGCTTCTTACCTAGTTCTGGGGGCAGAACG |
| circRSU1-Del1&3 | **F1:** TTTTTTTATACTTCAGTTACCTTATCCCATATCACACAACTGGTC |
|  | **R1:** GAGCACCTCCAAATTCTTCAGCGATGTTCGGTGGCACCATTG |
|  | **F2:** ATGGTGCCACCGAACATCGCTGAAGAATTTGGAGGTGCTCAACTT |
|  | **R2:** GGCAGCGAGATCAGGTCGTTAGGCTGAGTATCTGCAACTTTGTGAGC |
|  | **F3:** CAAAGTTGCAGATACTCAGCCTAACGACCTGATCTCGCTGCCTAAG |
|  | **R3:** TTTCCTTGCTTCTTACCTAGTTCTGGGGGCAGAACG |
| pCIR-circRSU1-P1 | **F1:** TTTTTTTATACTTCAGTTACCTTATCCCATATCACACAACTGGTC |
|  | **R1:** CATGTTTTCTGGAGTCGACCGAGCTCTTTAAGCTGGGTAAGCTCC |
|  | **F2:** ACCCATGTCTGCAGGTCGACCACATTCAGGGGAACCGCCTCA |
|  | **R2:** TTTCCTTGCTTCTTACCTAGTTCTGGGGGCAGAACG |
| pCIR-circRSU1-P2 | **F1:** TTTTTTTATACTTCAGTTACCTTATCCCATATCACACAACTGGTC |
|  | **R1:** CATGTTTTCTGGAGTCGACCCCTGTTCATGCCAAGGTTCAGGTG |
|  | **F2:** ACCCATGTCTGCAGGTCGACCTGAACACTTTGCCACGAGGCTT |
|  | **R2:** TTTCCTTGCTTCTTACCTAGTTCTGGGGGCAGAACG |
| 2XMS2 | **F:** GGTCGACTCCAGAAAACATGAGGATCACCCATGTCTGCAGTATTCCCGGGTTCAT  TAGATCCTAAGGTACCTAATTGCCTAGAAAACATGAGGATCACCCATGTCTGCAGGTCGAC |
|  | **R:** GTCGACCTGCAGACATGGGTGATCCTCATGTTTTCTAGGCAATTAGGTACCTTAG  GATCTAATGAACCCGGGAATACTGCAGACATGGGTGATCCTCATGTTTTCTGGAGTCGACC |
| Alu1 | **F:** AAAGTGCTGAGATTACAGGCGTGAGCCACCACCCCCGGCCCACTTTTTGTAAAGG  TACGTACTAATGACTTTTTTTTTATACTTCAG |
|  | **R:** CTGAAGTATAAAAAAAAAGTCATTAGTACGTACCTTTACAAAAAGTGGGCCGGGG  GTGGTGGCTCACGCCTGTAATCTCAGCACTTT |
| Alu2 | **F:** GTAAGAAGCAAGGAAAAGAATTAGGCTCGGCACGGTAGCTCACACCTGTAATCCC  AGCA |
|  | **R:** TGCTGGGATTACAGGTGTGAGCTACCGTGCCGAGCCTAATTCTTTTCCTTGCTTC  TTAC |
| HA-hnRNPA1 | **F:** GTCCCAGACTACGCTGCTGCTGTCGAATTC |
|  | **R:** ATAGGGCCCTCTAGATGCATGCTCGAG |
| HA-hnRNPA2B1 | **F:** TCGAATTCGAGAGAGAAAAGGAACAGTTCCGTAAGC |
|  | **R:** TGCTCGAGTCATTGGACCGTAGTTAGAAGGTTGC |
| HA-hnRNPA1-Δ14-97 | **F1:** GTCCCAGACTACGCTGCTGCTGTCGAATTC |
|  | **R1:** TTCACAGTTAAGTGGGCACCTGGCAGCTGTTCGGGCTCTTTAGGAGA |
|  | **F2:** TCTCCTAAAGAGCCCGAACAGCTGCCAGGTGCCCACTTAACTGTGAA |
|  | **R2:** ATAGGGCCCTCTAGATGCATGCTCGAG |
| HA-hnRNPA1-Δ105-184 | **F1:** GTCCCAGACTACGCTGCTGCTGTCGAATTC |
|  | **R1:** AAGCACTAGCCATCTCCACAGTTAAGTGGGCACCTGGTCT |
|  | **F2:** TGCCCACTTAACTGTGGAGATGGCTAGTGCTTCATCCAGCCA |
|  | **R2:** ATAGGGCCCTCTAGATGCATGCTCGAG |
| HA-hnRNPA1-Δ218-240 | **F1:** GTCCCAGACTACGCTGCTGCTGTCGAATTC |
|  | **R1:** CATTATAGCCATCCCCACCGAAGTTGTCATTCCCACCGA |
|  | **F2:** GAATGACAACTTCGGTGGGGATGGCTATAATGGATTTGGTAAATGATGGA |
|  | **R2:** ATAGGGCCCTCTAGATGCATGCTCGAG |
| HA-hnRNPA1-K8R | **F:** TCGAATTCCTAAGTCAGAGTCTCCTAGAGAGCCCGAACAGCTGAG |
|  | **R:** ATAGGGCCCTCTAGATGCATGCTCGAG |
| pBiCis -circRSU1-IRES | **F:** AGAGCGGCCGCAAGGCTTCGGCTCCCT |
|  | **R:** CCAAGGAGCTCCATATCTGCAACTTTG |

**Table S3: Sequence of ASOs and siRNAs used in this study.**

| **Oligos Name** | **Sequence** |
| --- | --- |
| CircRSU1-ASO#1 | GGTAACTAGTTCTGGGGGC |
| CircRSU1-ASO#2 | TGGGATAAGGTAACTAGTT |
| sihnRNPA1#1 | CAGCTGAGGAAGCTCTTCA |
| sihnRNPA1#2 | AATGGGGAACGCTCACGGACT |
| siHIF1A#1 | GCGAAGUAAAGAAUCUGAATT |
| siHIF1A#2 | GCUAUUCACCAAAGUUGAATT |
| siCD24#1 | CCCACGCAGAUUUAUUCCATT |
| siCD24#2 | GCACUAAUUUAAUGCCGAUTT |

**Table S4: PCR primers used in this study**

| **Primers Name** | **Sequence** |
| --- | --- |
| Human GAPDH | **F:** TCGACAGTCAGCCGCATCTTCTTT |
|  | **R:** ACCAAATCCGTTGACTCCGACCTT |
| Human 18S | **F:** GACTCAACACGGGAAACCTC |
|  | **R:** AGCATGCCAGAGTCTCGTTC |
| Human circRSU1 | **F:** GCCCCCAGAACTAGTTACCTT |
|  | **R:** TCAGTTCTGCGATGTTCGGT |
| Human circCSNK1G1 | **F:** GCCTCTTCGAAATCAGGTGAAG |
|  | **R:** CAAGTCCTCCAAGCTAGGGC |
| Human HNRNPA1 | **F:** GCCCTGTCAAAGCAAGAGATGG |
|  | **R:** CGACCACTGAAGTTTCCTCCAC |
| Human HIF1A | **F:** GAACGTCGAAAAGAAAAGTCTCG |
|  | **R:** CCTTATCAAGATGCGAACTCACA |
| Human HNRNPA2B1 | **F:** CAGCAACCTTCTAACTACGGTCC |
|  | **R:** CACTGCCTCCTGGACCATAGTT |
| Human MALAT1 | **F:** GAATTGCGTCATTTAAAGCCTAGTT |
|  | **R:** GTTTCATCCTACCACTCCCAATTAAT |
| Human AFP | **F:** GCAGAGGAGATGTGCTGGATTG |
|  | **R:** CGTGGTCAGTTTGCAGCATTCTG |
| Human CD24 | **F:** CTCCTACCCACGCAGATTTATTC |
|  | **R:** AGAGTGAGACCACGAAGAGAC |
| Human RSU1 | **F:** TACCTCTACGGCAGACACATGCA |
|  | **R:** CACACGCAGCATTGGGTTTATTT |
| Human U6 | **F:** CTCGCTTCGGCAGCACA |
|  | **R:** AACGCTTCACGAATTTGCGT |
| hsa-miR-885-3p | AGGCAGCGGGGTGTAGTGGATA |
| hsa-miR-767-5p | TGCACCATGGTTGTCTGAGCATG |
| hsa-miR-335 | CGGTCAAGAGCAATAACGAAAAATGT |
| Mouse Cd24 | **F:** CATCTGTTGCACCGTTTCCC |
|  | **R:** CTGGTGGTAGCGTTACTTGGA |

**Supplementary Figures**


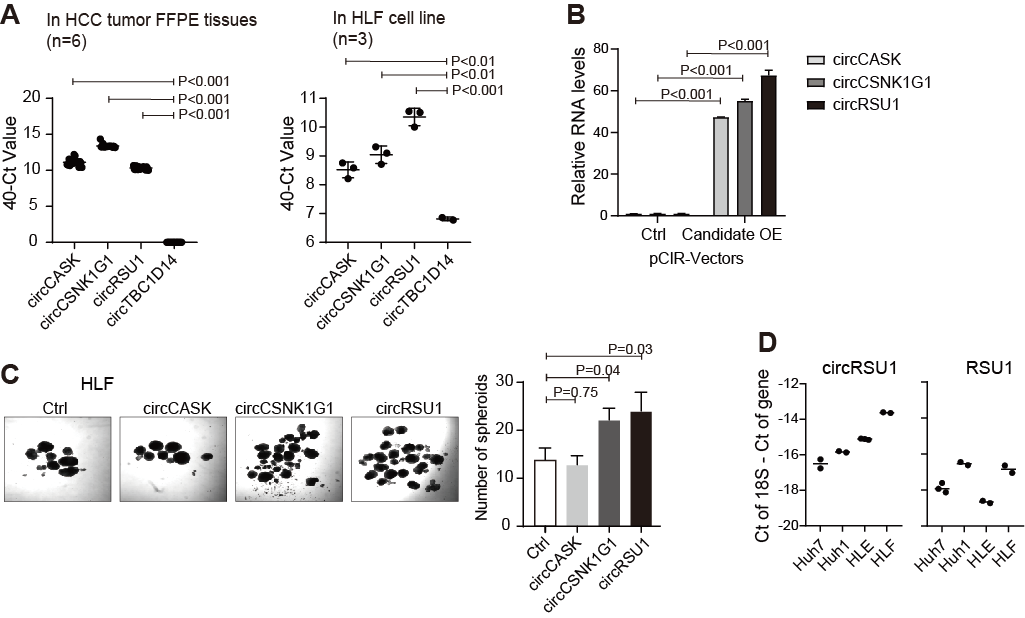


**Figure S1. Identification of circRNAs with high levels in tumors and abilities of promoting stemness features in HCC.**

(A) The expression levels of four candidate circRNAs in HCC tumor FFPE tissues from Cohort 1 (n=6) and in HCC HLF cell line. (B) Overexpression efficiency of the constructed circRNA vectors in the HLF cell line. (C) Spheroid formation in HLF cells transfected with control or circRNA vectors. Student’s t-test was performed. (D) Expression levels of circRSU1 and linear RSU1 across different hepatic cell lines. Data are shown as −ΔCt values (Ct_18s_ - Ct_gene_ ). (A-C) Student’s t-test was performed.


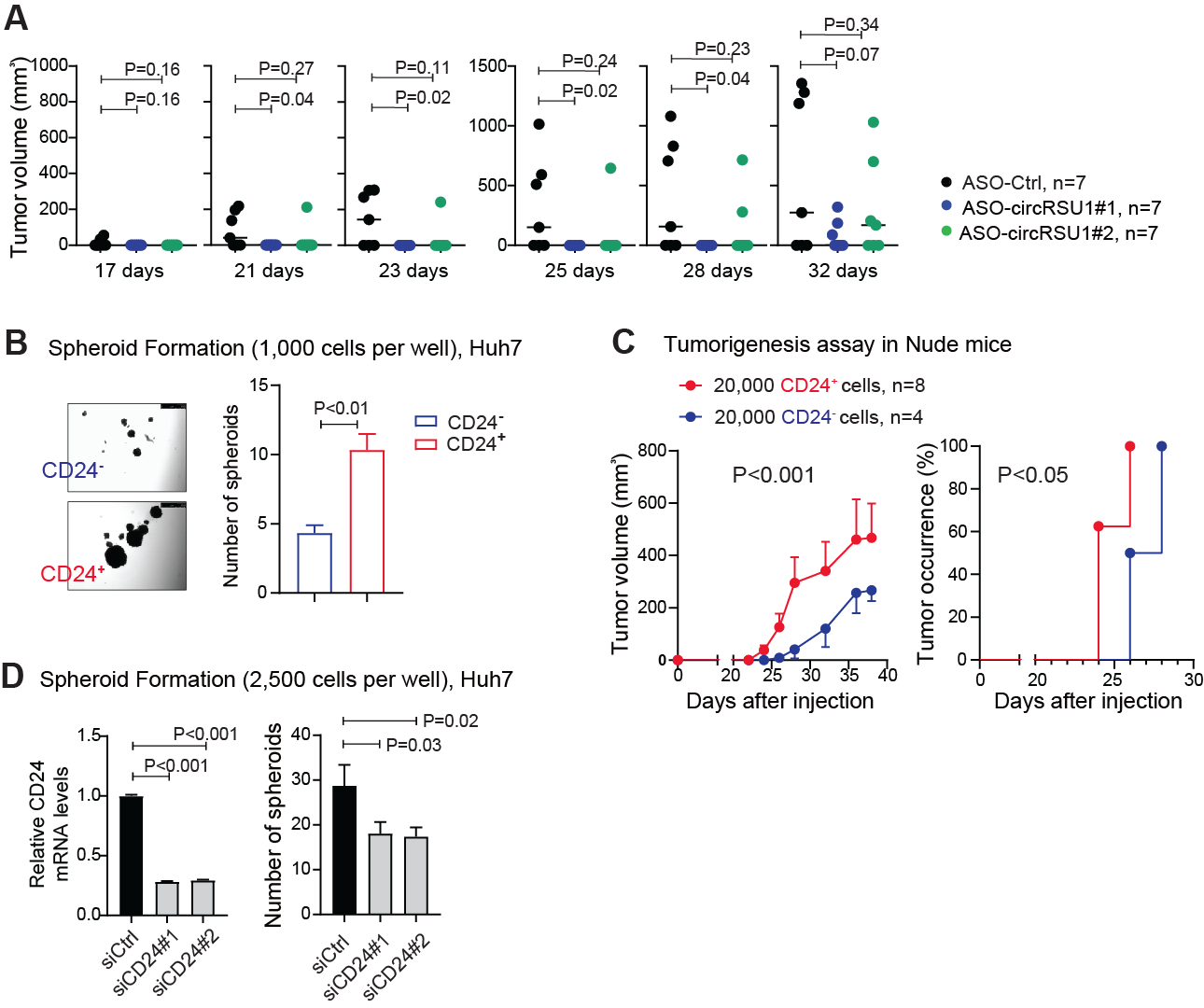


**Figure S2. The cancer stem cell features of CD24^positive^ cells.**

(A) Individual tumor volumes measured at each indicated time point in Huh7 xenograft models treated with ASO-Ctrl or ASO-circRSU1 (#1 or #2). Each dot represents one tumor site from an individual mouse (7 sites/ group). (B) Spheroid formation of the sorted CD24^positive^ and CD24^negative^ Huh7 cells. Cells were seeded at 1,000 cells per well in low-attachment plates. Data are shown as mean ± SD. (C) Tumorigenicity assay was performed with 20,000 CD24^positive^ and CD24^negative^ Huh7 cells in male BALB/c nude mice. The two-way ANOVA analysis was performed. (D) Spheroid formation assay of Huh7 cells after CD24 knockdown. Cells were seeded at 2,500 cells per well in low-attachment plates. Student’s t-test (A, B, D) and two-way ANOVA (C) were used for statistical analysis.


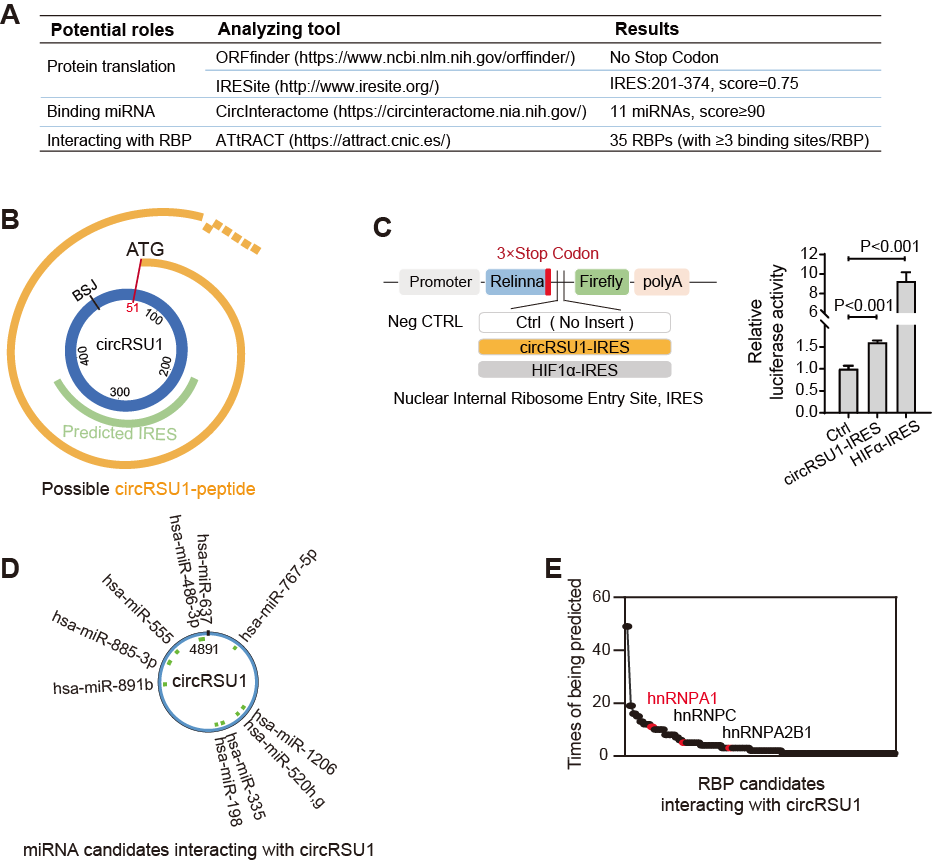


**Figure S3. The potential of circRSU1 in coding proteins or working as miRNA sponges.**

(A) Predicted molecular biological functions of circRSU1 via different online prediction tools. (B) Schematic figure of the potential peptides encoded by circRSU1, predicted from the ORF Finder and IRESite online databases. (C) Diagram of pBiCis-Ctrl, pBiCis-circRSU1-IRES, and pBiCis-HIF1A -IRES dual-luciferase reporter constructs (left panel), and dual-luciferase reporter assay of the IRES activity of the predicted circRSU1 IRES sequence (right panel). *HIF1A* IRES was used as a positive control. Student’s t-test was used. (D) The predicted candidate miRNAs interacting with circRSU1 and their interaction sites. (E) AtTRACT prediction of RBPs potentially interacting with circRSU1, with the y-axis representing the frequency of each RBP in the prediction list.


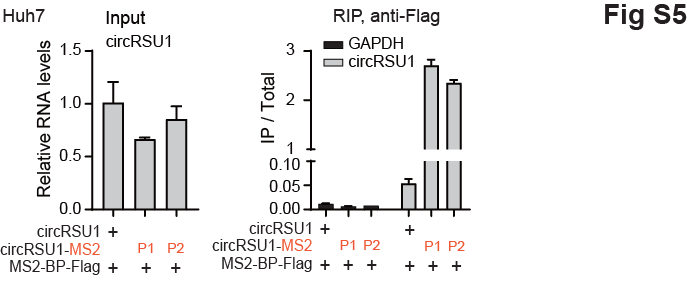


**Figure S4. Validation of the MS2 system in circRSU1 enrichment.**

Huh7 cells were transfected with plasmids overexpressing circRSU1-WT, circRSU1-MS2-P1, or circRSU1-MS2-P2, along with MS2-BP-Flag. The enrichment of circRSU1 was assessed by RT-qPCR following MS2-Flag immunoprecipitation using anti-Flag beads. GAPDH was used as the control RNA.


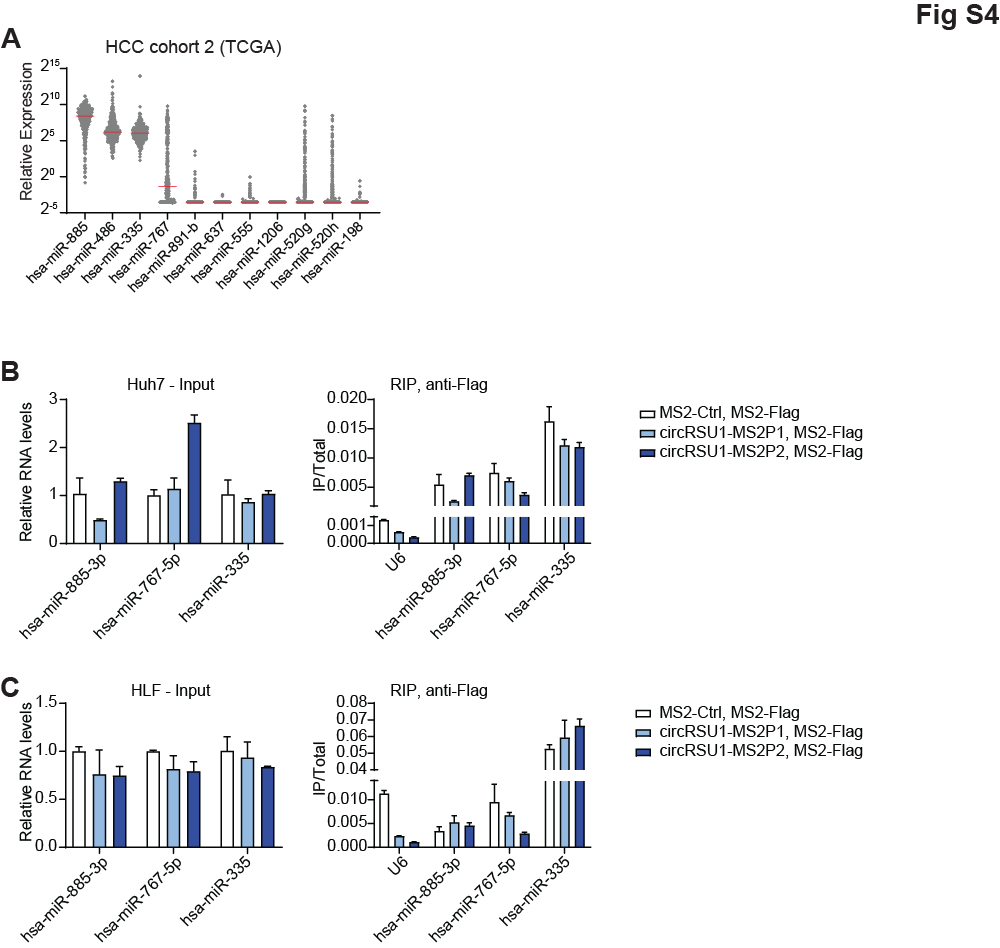


**Figure S5. miRNA expression and miRNA enrichment by circRSU1.**

(A) Expression levels of 11 miRNA candidates in HCC cohort 2. Among them, three out of 11 miRNAs had the relatively high abundance. (B-C) Enrichment of the four miRNA candidates in Huh7 and HLF cell lines in MS2 RIP assay. miR-486 was under detected and the rest three were shown. U6 was used as the control RNA for the miRNA detection.


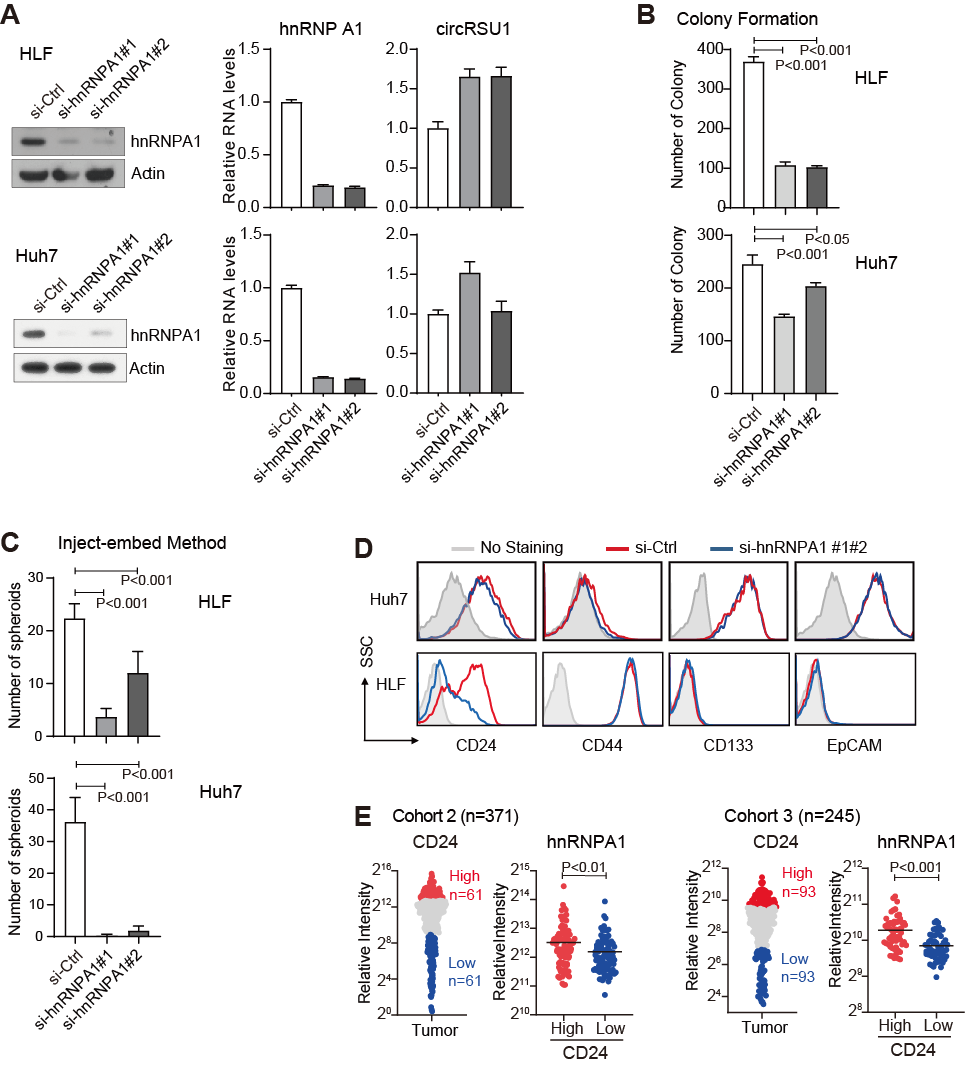


**Figure S6. Silencing hnRNPA1 significantly suppresses the malignant phenotype of HCC.**

(A) hnRNPA1 silencing efficiency and its effect on circRSU1 expression level in HLF and Huh7 cell lines. (B) Colony formation in HLF and Huh7 cells transfected with si-Ctrl or si-hnRNPA1. (C) Spheroid formation with inject-embed method in HLF and Huh7 cells transfected with si-Ctrl or si-hnRNPA1. (D) Flow cytometry was performed to assess the proportion of cells positive for CSC markers (CD24, CD44, CD133, and EpCAM) in Huh7 and HLF cells transfected with si-Ctrl or si-hnRNPA1. (E) HCC patients were stratified into CD24^positive^ and CD24^negative^ groups based on the tertile cut-off of CD24. The hnRNPA1 RNA level was compared between the CD24^positive^ and CD24^negative^ patient groups. (B, C, E) Statistical analysis was performed using Student’s t-test.


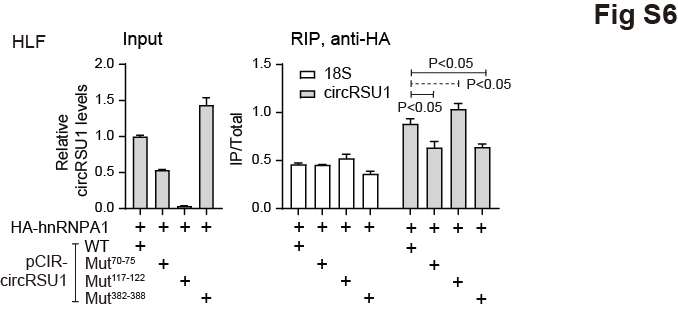


**Figure S7. Enrichment of circRSU1 in RIP assay using anti-HA beads.**

The assay was performed in HLF cells transfected with HA-hnRNPA1 and various circRSU1 vectors. 18S was used as the control RNA. Student’s t-test was used for statistical analysis.


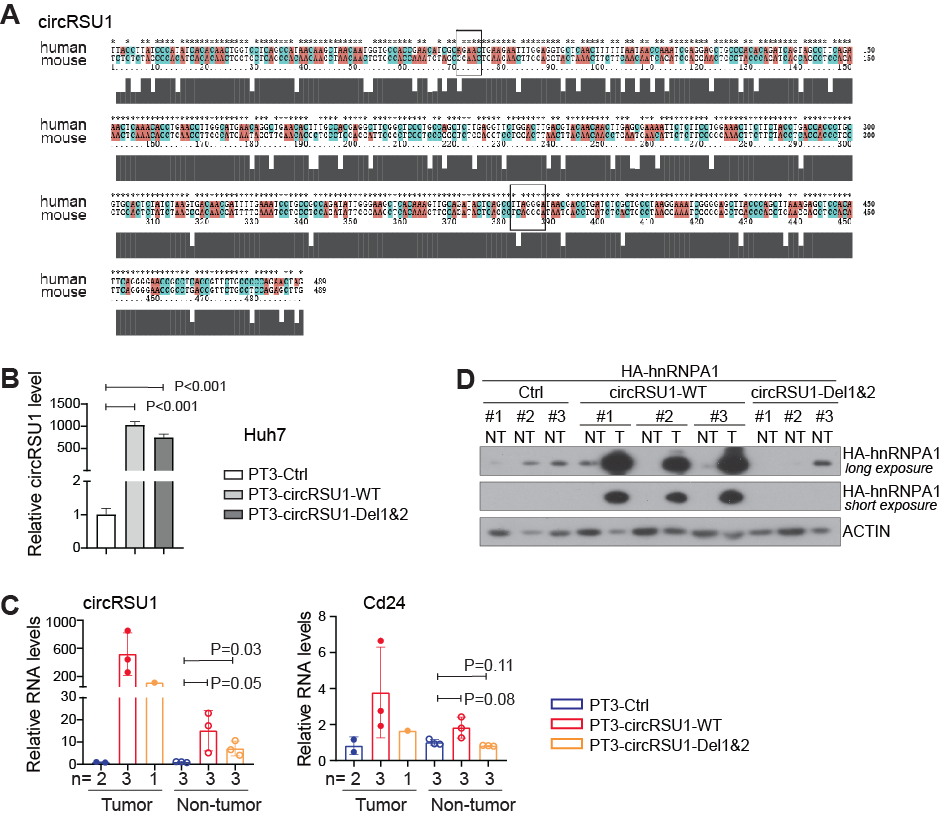


**Figure S8. circRSU1 and hnRNPA1 expression efficiency in orthotopic tumorigenesis model.**

(A) Sequence conservation analysis of circRSU1 between human and mouse. The two black square indicate the two interacting motifs of circRSU1 with hnRNPA1. (B) Expression efficiency of the PT3-circRSU1-WT and Del1&2 vectors in Huh7 cells. (C) Detection of circRSU1 and *Cd24* expression in tumor (T) and non-tumor (NT) tissues among three groups. Tissue numbers are indicated. Student’s t-test was used for statistical analysis. (D) Western Blot analysis of hnRNPA1 expression in available tumor and non-tumor liver tissues from the orthotopic model. (B-C) Student’s t-test was used for statistical analysis.


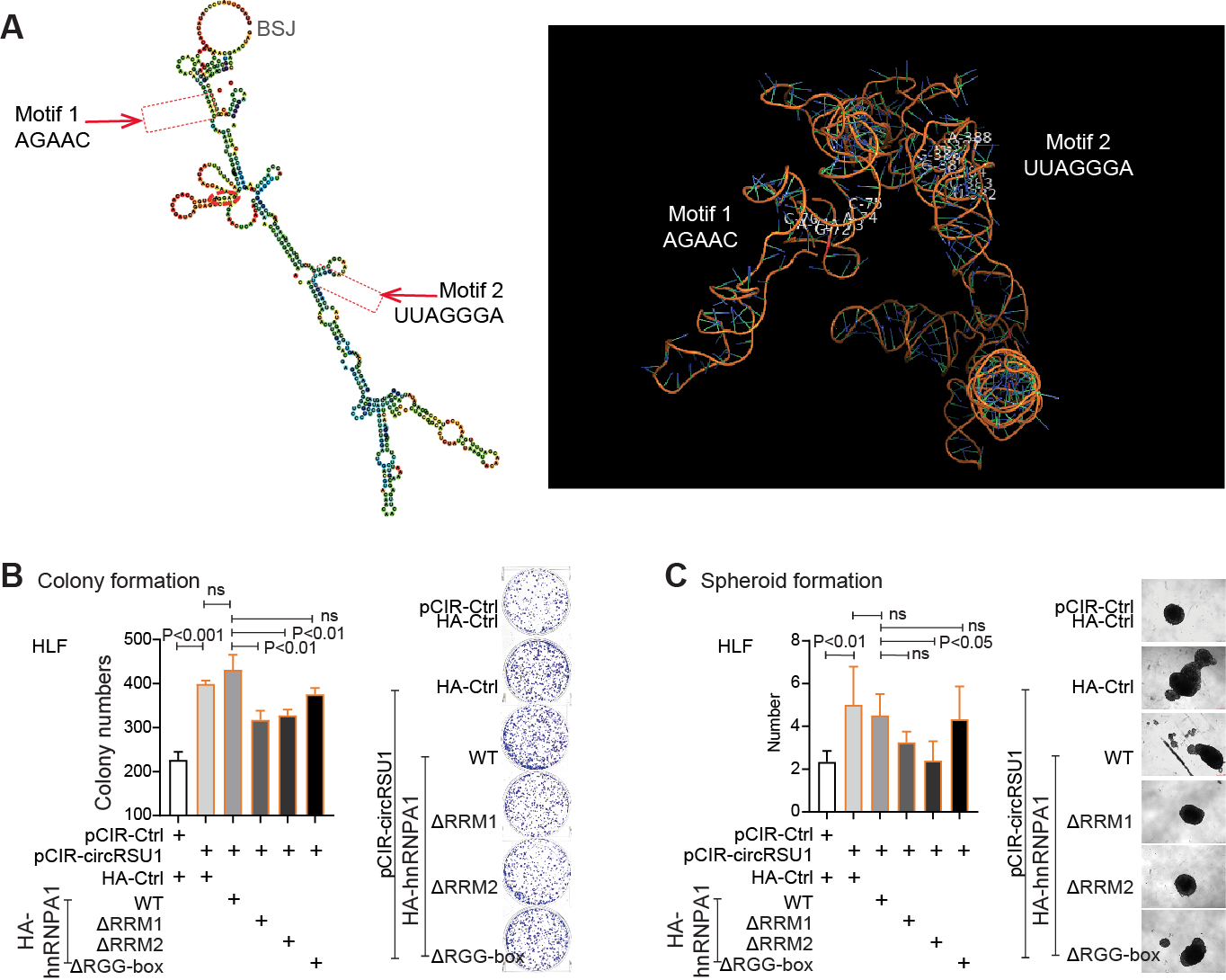


**Figure S9. The predicted spatial folding of circRSU1 and phenotypes of HLF cells co-expressing circRSU1 and different truncated forms of HA-hnRNPA1.**

(A) The predicted spatial folding of circRSU1 via the online tool RNAComposer (<https://rnacomposer.cs.put.poznan.pl/>). The two hnRNPA1-interacting domains were labeled. (B-C) Colony formation (B) and spheroid formation (C) with low-attachment plates with HLF cells co-expressing circRSU1 and different truncated forms of HA-hnRNPA1. Student’s t-test was performed.


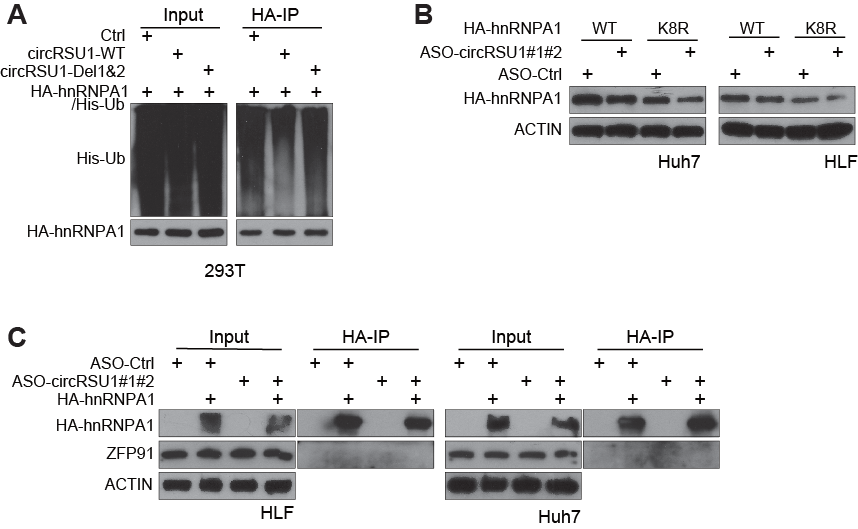


**Figure S10. The effect of circRSU1 on the ubiquitination of hnRNPA1.**

(A) The polyubiquitination levels of hnRNPA1 were assessed in 293T cells transfected with circRSU1-WT or circRSU1-Del1&2 along with HA-hnRNP A1 and His-Ub plasmids. IP was performed using anti-HA beads. (B) HnRNPA1 protein level in Huh7 and HLF cells transfected with ASO-Ctrl or ASO-circRSU1 along with HA-hnRNPA1-K8R or HA-hnRNPA1 vectors. (C) The immunoprecipitation was performed to examine the interaction levels of hnRNPA1 and ZFP91 in Huh7 and HLF cells transfected with HA-hnRNP A1, along with ASO-Ctrl or ASO-circRSU1.

**
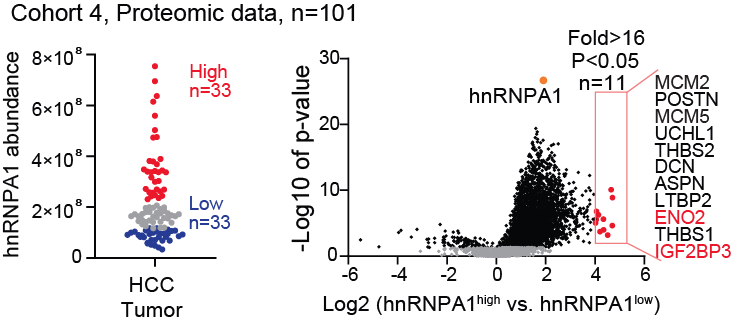
**

**Figure S11. Class comparisons of proteome between HCCs with high hnRNPA1 and HCCs with low hnRNPA1 protein level.**

Differentially expressed proteins between hnRNPA1^high^ and hnRNPA1^low^ groups were analyzed in HCC Cohort 4. Proteins with significant high expression in hnRNPA1^high^ group were labeled in Red and listed. Proteins in red color refer to the known HIF-1α regulator or target.


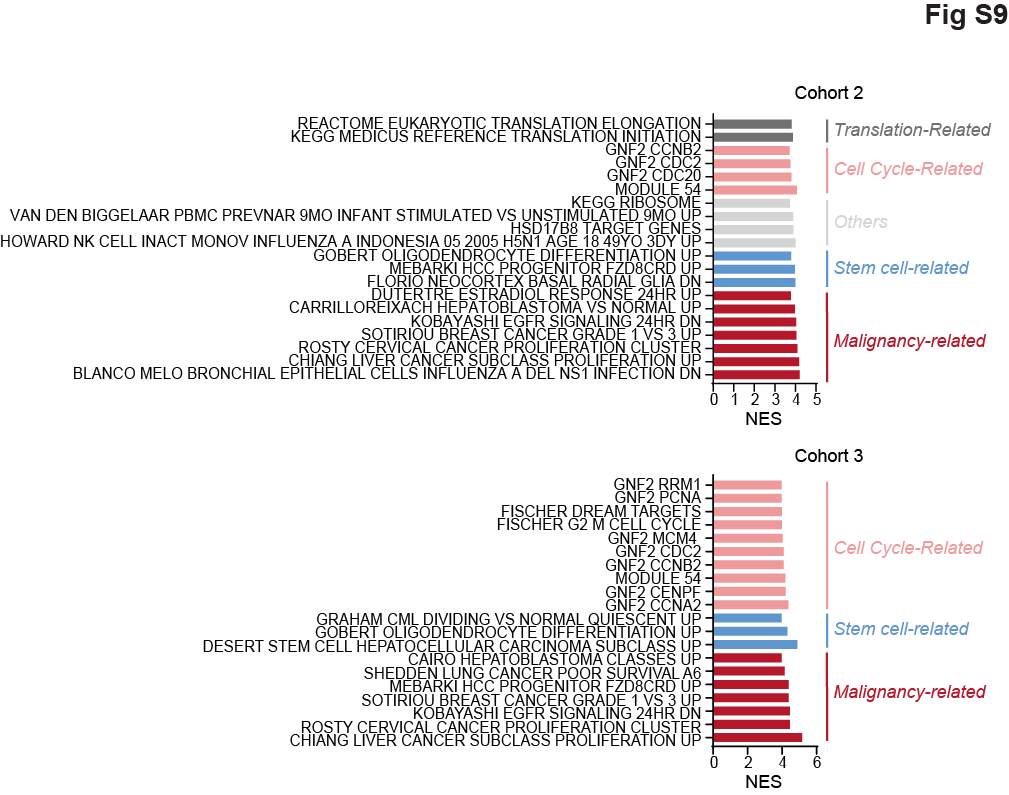


**Figure S12. The top 20 enriched signatures in hnRNPA1^high^ group of HCC cohorts 2-3.**

Red bars refer to malignancy-related signatures. Blue bars refer to stem cell-related signatures. Pink bars refer to cell cycle-related signatures. Dark grey bars refer to translation-related signatures and light grey bars refer to other signatures.

**
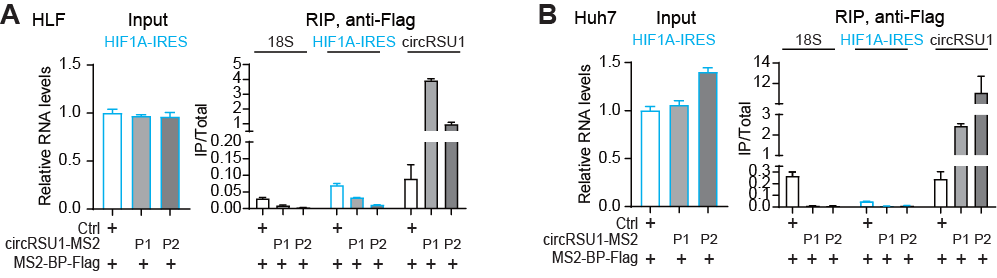
**

**Figure S13. circRSU1 does not directly bind to *HIF1A* IRES.**

Enrichment of *HIF1A* IRES was assessed by RT-qPCR following MS2-Flag RIP assay using anti-Flag beads in Huh7 (A) and HLF (B) cells. Cells were co-transfected with various circRSU1-MS2 and MS2-Flag. 18S was used as the control RNA.


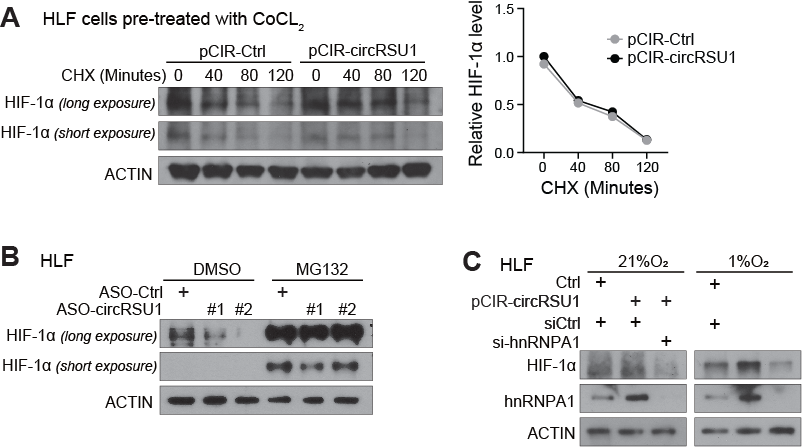


**Figure S14. circRSU1 does not enhance HIF-1α protein stability.**

(A) HIF-1α protein stability via CHX chase assay in HLF cells transfected with control vector or pCIR-circRSU1. Cells were pretreated with CoCl₂ for 36 hours to allow HIF-1α accumulation, followed by CHX treatment to block de novo protein synthesis for the indicated time. (B) HIF-1α protein levels in HLF cells transfected with ASO-Ctrl or ASO-circRSU1 and under MG132 treatment. (C) HIF-1α protein levels in HLF cells transfected with control vector or pCIR-circRSU1 under normoxic (21% O₂) or hypoxic (1% O₂) conditions, with or without hnRNPA1 knockdown.


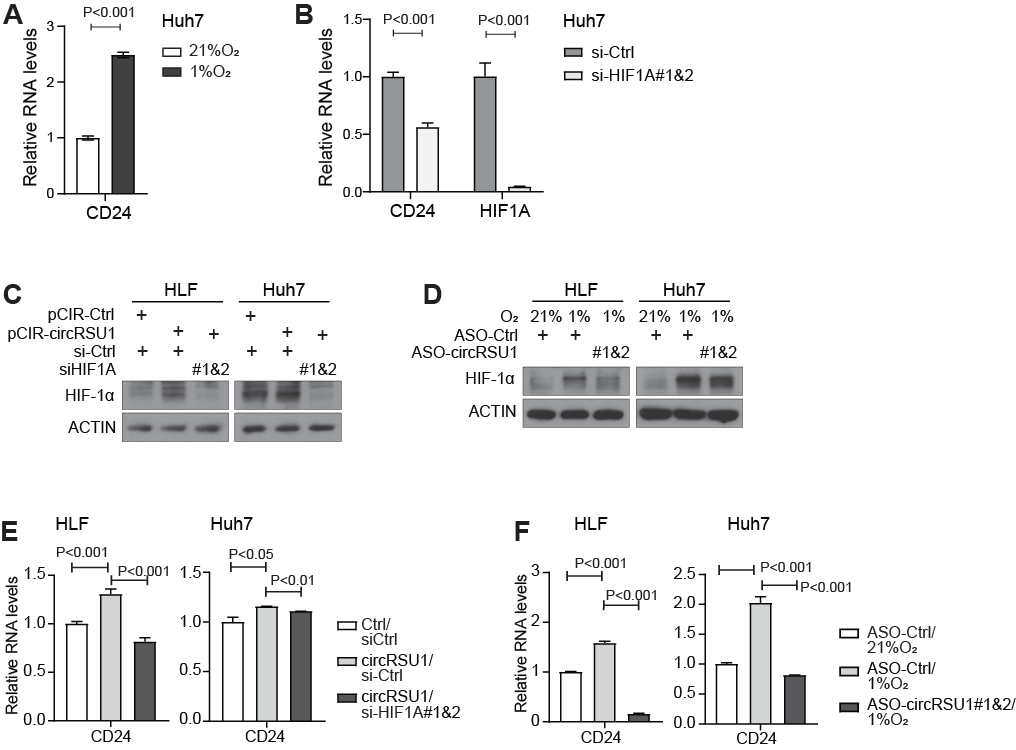


**Figure S15. The effects of *HIF1A* knockdown and hypoxic condition on CD24 and HIF-1α.**

(A) qRT-PCR analysis of *CD24* expression in Huh7 cells cultured under normoxic (21% O₂) or hypoxic (1% O₂) conditions. Data are shown as mean ± SD. (B) qRT-PCR analysis of *CD24* and *HIF1A* expression in Huh7 cells transfected with control siRNA or siRNAs targeting *HIF1A*. Data are shown as mean ± SD. (C) HIF-1α protein level in HLF and Huh7 cells transfected with control, circRSU1, or circRSU1 combined with HIF-1α knockdown. (D) HIF-1α protein level in HLF and Huh7 cells transfected with ASO-Ctrl or ASO-circRSU1 under normoxia or hypoxia conditions. (E) RT-qPCR analysis of *CD24* level in HLF and Huh7 cells transfected with control, circRSU1, or circRSU1 combined with HIF-1α knockdown. Student’s t-test was performed. (F) RT-qPCR analysis of *CD24* expression level in HLF and Huh7 cells transfected with ASO-Ctrl or ASO-circRSU1 under normoxia or hypoxia conditions. (A, B, E, F) Student’s t-test was performed.
